# Unveiling the microbial diversity across the northern Ninety East Ridge in the Indian Ocean

**DOI:** 10.3389/fmicb.2024.1436735

**Published:** 2024-09-24

**Authors:** Ding Li, Liping Wang, Fan Jiang, Xiang Zeng, Qinzeng Xu, Xuelei Zhang, Qiang Zheng, Zongze Shao

**Affiliations:** ^1^Key Laboratory of Marine Genetic Resources, Third Institute of Oceanography, Ministry of Natural Resources of PR China, State Key Laboratory Breeding Base of Marine Genetic Resources, Fujian Key Laboratory of Marine Genetic Resources, Xiamen, China; ^2^State Key Laboratory for Marine Environmental Science, Institute of Marine Microbes and Ecospheres, College of Ocean and Earth Sciences, Xiamen University, Fujian Key Laboratory of Marine Carbon Sequestration, Xiamen, China; ^3^First Institute of Oceanography, Ministry of Natural Resources of PR China, Qingdao, Shandong, China

**Keywords:** Ninety-East Ridge, microbial diversity, 16S rRNA gene, ecological differentiation, bioindicator

## Abstract

Prokaryotes play a crucial role in marine ecosystem health and drive biogeochemical processes. The northern Ninety East Ridge (NER) of the Indian Ocean, a pivotal yet understudied area for these cycles, has been the focus of our study. We employed high-throughput 16S rRNA gene sequencing to analyze 35 water samples from five stations along the ridge, categorized into three depth- and dissolved oxygen-level-based groups. Our approach uncovered a clear stratification of microbial communities, with key bioindicators such as *Prochlorococcus* MIT9313, Sva0996 marine group, and *Candidatus Actinomarina* in the upper layer; *Ketobacter*, *Pseudophaeobacter*, *Nitrospina*, and SAR324 clade in the middle layer; and *Methylobacterium-Methylorubrum*, *Sphingomonas*, *Sphingobium*, and *Erythrobacter* in the deep layer. *Methylobacterium-Methylorubrum* emerged as the most abundant bacterial genus, while *Nitrosopumilaceae* predominated among archaeal communities. The spatial and depth-wise distribution patterns revealed that *Ketobacter* was unique to the northern NER, whereas *Methylobacterium-Methylorubrum*, UBA10353, SAR324 clade, SAR406, Sva0996_marine_group, *Candidatus Actinomarina* were ubiquitous across various marine regions, exhibiting niche differentiation at the OTU level. Environmental factors, especially dissolved oxygen (DO), silicate, nitrate, and salinity, significantly influence community structure. These findings not only reveal the novelty and adaptability of the microbial ecosystem in the northern NER but also contribute to the broader understanding of marine microbial diversity and its response to environmental heterogeneity.

## 1 Introduction

The world’s oceans host a multitude of unique and intricate ecosystems, ranging from ridges to basins, cold seeps, hydrothermal vents, and oxygen minimum zones (OMZs) (Wei et al., 2008; Baumgartner et al., 2022; Chen et al., 2022; Li et al., 2022). These diverse habitats harbor distinct microbial communities that play essential roles in regulating oceanic biogeochemical cycles, thereby significantly contributing to the overall health and function of the marine environment. The composition and structure of these microbial communities are influenced not only by their unique habitats but also by various ecological processes, such as dispersal, recombination, and coevolution, which are driven by interactions such as symbiosis and competition (Wei et al., 2008; Fernandes et al., 2019; Zhang et al., 2022; Dong et al., 2023).

Previous studies have highlighted the concept of vertical zonation within microbial communities. This stratification is particularly evident in underwater plateaus such as the Benham Rise in the northeastern Philippines, where different layers of the water column support different microbial populations (DeLong et al., 2006; Hewson et al., 2006; Gajigan et al., 2018). The physical separation of the water column presents a barrier to microbial dispersal, leading to distinct layers of microbial life. Comparative studies across different oceanic regions, including the Indian and Pacific Oceans, have revealed a connectivity between surface and deep-water bacterial populations, suggesting that certain surface bacteria can also thrive in the deep sea (Sow et al., 2022). Furthermore, biogeographic partitioning among microbial communities has been observed in various regions of the Indian Ocean, including its southwestern, central, and Bay of Bengal areas. This indicates a fine-scale spatial separation of bacterial populations, which is likely a result of the interplay between environmental conditions and microbial adaptations (Jeffries et al., 2015; Zheng and Huang, 2016; Raes et al., 2018; Hörstmann et al., 2021).

The distribution and structure of bacterial communities in both water and sediment environments are influenced by numerous factors (Bouvier and del Giorgio, 2002; Zheng et al., 2013; Guo et al., 2018). Parameters such as fluorescence, nitrite, phosphate, silicate, and depth, are crucial in shaping the composition of bacterial and archaeal communities in the South Atlantic’s epipelagic zone (Ferreira et al., 2022). Dissolved oxygen (DO) has been recognized as a critical factor, particularly impacting the distribution of anammox bacteria in the deep seas of the Eastern Indian Ocean (Qian et al., 2018). These factors collectively shape the distribution and structure of microbial communities, which in turn affect the ecosystem’s overall health and function.

To date, the microbial diversity of the Indian Ocean’s water column has been extensively investigated, much of this focus has been on microbes involved in nitrogen cycling near OMZs, with comparatively less attention given to other habitats within the water column (Long et al., 2021). Studies have identified ubiquitous and cosmopolitan taxa such as *Actinobacteria*, *Bacteroidetes*, *Cyanobacteria*, SAR406 clade, *Proteobacteria*, and *Verrucomicrobia* in the Bay of Bengal and the Indian Ocean abyssal regions (Wang et al., 2016; Barnes et al., 2021). However, despite the acknowledged importance of these microbes, in the Indian Ocean, the composition of microbial communities in the Northern Ninety East Ridge has been largely overlooked, resulting in a gap in our understanding of their ecological dynamics and the biogeochemical processes they mediate. The NER’s unique geological and hydrographic features likely harbor distinct microbial communities, contributing to specific biogeochemical processes.

The Ninety East Ridge (NER), extending over 5,600 km in a south–north orientation, is marked by a complex array of geological and oceanographic features. These include tectonics, topography, bathymetry, and hydrography, which likely influence the distribution and diversity of the region’s microbial inhabitants (Sclater and Fisher, 1974; Bandekar et al., 2018; Gao et al., 2020). Although the microbial diversity of the NER has been partially explored, with studies often focusing on either deep or surface water layers, a cohesive understanding of the region’s prokaryotic communities and their ecological roles has yet to be realized. Recent studies on the central NER have reported a higher abundance and diversity of bacteria relative to archaea and fungi, with the dominant microbial groups varying by depth and locations. Factors like depth, DO, and nitrite have been identified to significantly influence the structuring of bacterial communities in the central NER (Gao et al., 2020). Additionally, the identification of indicator species within the water masses of the southern NER suggests their potential as biogeographic markers for these regions (Li et al., 2022).

Despite these advances, a comprehensive synthesis of the NER’s prokaryotic communities and their ecological roles is lacking. This study aims to address this knowledge gap by conducting a comprehensive investigation of prokaryotic communities across the northern NER. A total of 35 water samples were collected from five stations within the northern NER, encompassing depths from the surface to the Deep Chlorophyll Maximum (DCM) layer, the OMZs layer, and the deep and bottom layers down to 4,000 m.

Using Illumina 16S rRNA gene sequencing, we characterized the diversity and distribution of bacterial and archaeal communities across the water column, in relation to physicochemical variables. Our goal was to elucidate the unique bacterial and archaeal assemblages, assess their correlations with environmental parameters, and highlight the ecological niche differentiation of indicator species within the northern NER and other marine regions. The findings of this study are expected to enhance the understanding of microbial diversity in the northern NER and provide a foundation for future research into the functional roles and adaptive strategies of these communities in the biogeochemical cycling.

## 2 Materials and methods

### 2.1 Sample collection and methodology

**Figure 1:**
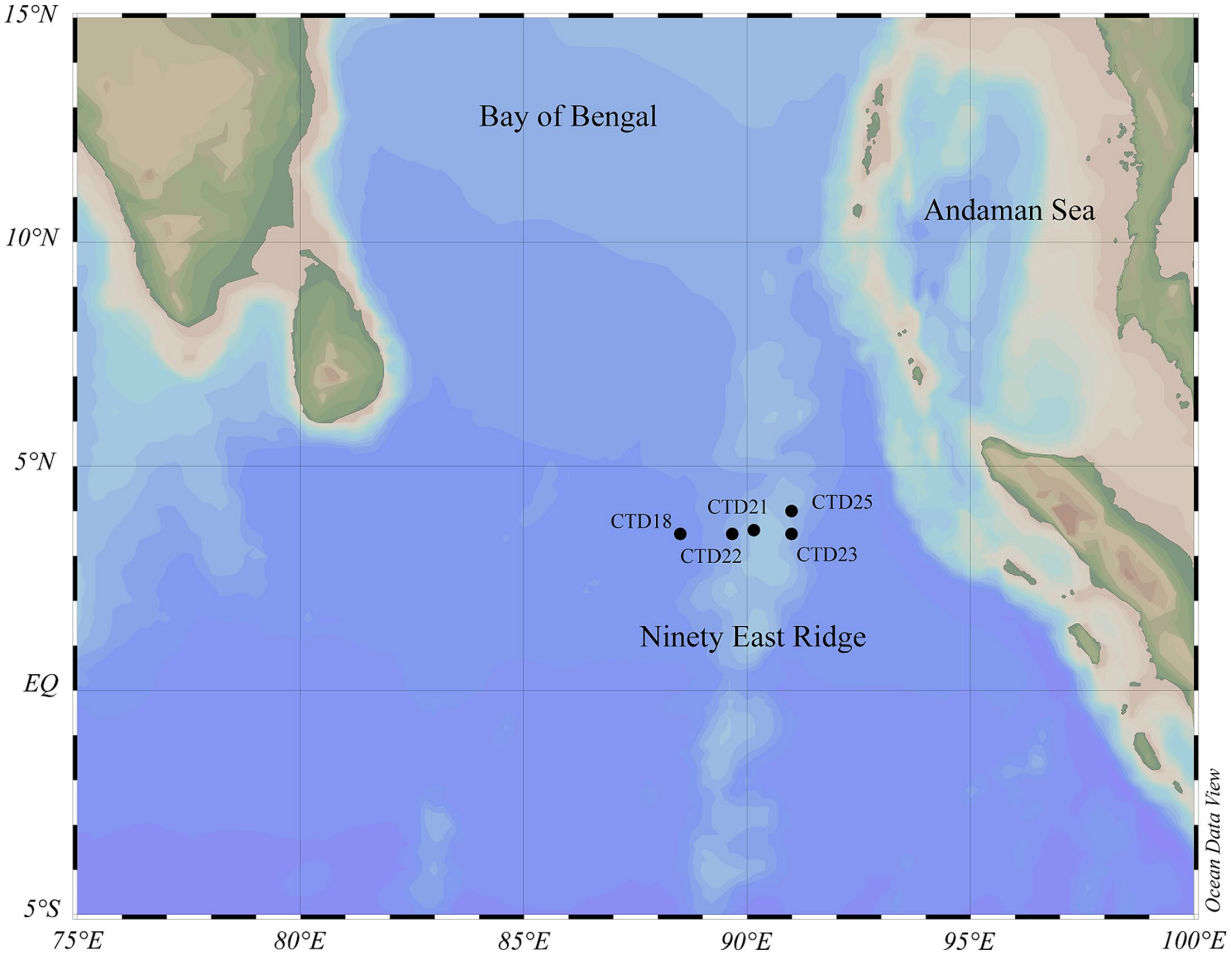
The geographic locations of 5 sampling stations in the Ninety-East Ridge (NER). Black solid circles represent the investigation stations.

Seawater samples were collected from 5 stations located along the Ninety-East Ridge of northern Indian Ocean during “China DY72 cruise” onboard the Research Vessel “Shen Hai Yi Hao.” The NER (latitude: 3.499556°N-4.000058°N, longitude: 88.500955°E-91.000289°E, water depth: 2232 m–4212 m) located in the northern Ninety East Ridge, was sampled from 26th June 2022 to 10th July 2022. For most stations, the sampling depths included DCM, OMZs, 1,000 m, 2,000 m, and the bottom. In total, 35 samples from 5 stations were collected from NER. These samples were categorized into three groups based on depth and dissolved oxygen levels, including the upper water layer (NER_UP), middle water layer (NER_MID), and deep water layer (NER_DOWN). The detailed information of sampling sites and depths is provided in Figure 1 and Supplementary Table 1.

Seawater samples were collected using a Conductivity-Temperature-Depth (CTD) rosette system fitted with 24 Niskin bottle samplers (Seabird Electronics, Washington, NY, United States), each equipped with an oxygen sensor (Aravamudhan et al., 2005). Eight liters of seawater were filtered through 0.22 μm Sterivex filters (Millipore, United States). The filter samples were immediately frozen in liquid nitrogen and stored at −80°C until DNA extraction. Nutrient parameters, including silicate, phosphate, nitrate, nitrite, and ammonium, were measured using a Skalar Autoanalyser (Skalar Analytical, Netherlands), following the standard methods.

### 2.2 Total genomic DNA extraction and quality inspection

Eight liters of seawater was filtered through a filter membrane and the DNA was extracted from the collected samples. The genomic DNA was isolated using DNeasy PowerSoil Pro Kit, following the manufacturer’s protocol (QIAGEN, Germany).^1^ The concentration of the extracted DNA was measured using a Qubit 3.0 Fluorometer (Life Technologies, ThermoFisher Scientific, USA) and the Equalbit 1x dsDNA HS Assay Kit (Vazyme, China).^2^ Prior to sequencing, the quality and concentration of the DNA were determined again by 1.0% agarose gel electrophoresis and a NanoDrop® ND-2000 spectrophotometer (Thermo Scientific Co., Ltd., USA).

### 2.3 Amplification of the 16S rRNA gene

The primer set 338F (5′-ACTCCTACGGGAGGCAGCAG-3′) and 806R (5′-GGACTACHVGGGTWTCTAAT-3′) was used to amplify the V3-V4 region of bacterial 16S rRNA gene, and the primer sequences were based on previous studies (Microbial population part) (Liu et al., 2016b). The primer set 524F10extF (5′- TGYCAGCCGCCG CGGTAA- 3′) and Arch958RmodR (5′- YCCGGCGTTGAVTCC AATT- 3′) was used to amplify the V4-V5 region of archaeal 16S rRNA gene, and the primer sequences were based on previous studies (2.2. DNA extraction, PCR and Illumina sequencing part) (Liu et al., 2016a).

The PCR reaction was conducted in a 20 μL reaction volume, including 10 ng of DNA, 0.8 μL each of primers (10 μM), 4 μL 5x FastPfu buffer, 0.4 μL FastPfu polymerase, 0.2 μL BSA, and 250 mM dNTPs (TransGen, China).^3^ For bacterial 16S rRNA gene amplification, PCR cycling conditions were as follows: initial denaturation at 95°C for 3 min, followed by 29 cycles of denaturation at 95°C for 30 s, annealing at 55°C for 30 s, extension at 72°C for 45 s and a final extension at 72°C for 10 min. For archaeal 16S rRNA gene amplification, PCR cycling conditions were as follows: initial denaturation at 95°C for 3 min, followed by 35 cycles of denaturation at 95°C for 30 s, annealing at 55°C for 30 s, extension at 72°C for 45 s, and a final extension at 72°C for 10 min.

### 2.4 Illumina sequencing and bioinformatic analysis

Purified amplicons were combined in equimolar proportions and sequenced using an Illumina MiSeq PE300 platform (Illumina, San Diego, USA), following the standard protocols provided by Majorbio Bio-Pharm Technology Co. Ltd. (Shanghai, China). Additionally, the data from others’ work was used Illumina Novaseq platform and Illumina Hiseq2500 platform (Beman et al., 2021; Gu et al., 2022; Li et al., 2022; Li et al., 2024).

The QIIME 2 pipeline was utilized for processing raw reads and downstream analysis (Bokulich et al., 2018; Bolyen et al., 2019). In brief, raw sequence data were demultiplexed and primer-trimmed, followed by quality filtering, denoising, merging, and chimera removal using the DADA2 plugin (Martin, 2011; Callahan et al., 2016). Non-singleton amplicon sequence variants (ASVs) were aligned with MAFFT and used to construct a phylogeny with FastTree2 (Price et al., 2009; Katoh et al., 2002). Alpha diversity metrics, encompassing Sobs (number of observed species), Shannon index (measuring microbial diversity in the sample), and Coverage (species coverage), as well as beta diversity metrics (Jaccard distance), were computed using Mothur v1.30.1 (Schloss et al., 2009). Taxonomy assignment was performed using the classify-sklearn naïve Bayes taxonomy classifier against the SILVA Release 138 Database (bacteria and archaea).

The differences in microbial composition and ecological differentiation of key taxa in five marine regions of northern NER (this study), central NER (14°S-18°S, 86°E-90°E), Arabian Sea (10°N-17°N, 63°E-68°E), ETNP (14°N-28°N, 105°W-115°W), Bay of Bengal (typical hypoxic zone, 15°N-2°N, 85°E-95°E) and were compared. Publicly accessible 16S rRNA gene high-throughput sequencing data were obtained from the National Center for Biotechnology Information (NCBI) for the cNER, ETNP and BoB. This dataset encompassed 20 samples from the surface layer (2 m), DCM, 500 m layer and 2000 m layers of the BoB, 25 samples from the surface layer (5 m), 50 m layer, 100 m layer, 200 m layer and 2000 m depth layers of the cNER, 40 samples from the surface layer (5 m), 50 m layer, DCM, OMZ, 500 m layer, 1000 m layer, 2000 m layer and Bot layers of the ETNP, as well as 37 samples from the surface layer (5 m) to 4000 m depth layers of the AS. The primers used in the studies of BoB and cNER were 341F (5′-CCTAYGGGRBGCASCAG-3′) and 806R (5′-GGACTACNNG GGTATCTAAT-3′), and the primers used in the studies of ETNP were 515F-Y (5′-GTGYCAGCMGCCGCGGTAA-3′) and 926R (5′-CCG YCAATTYMTTTRAGTTT-3′), the primers used in the studies of AS were consistent with this study (Beman et al., 2021; Gu et al., 2022; Li et al., 2022; Li et al., 2024). To enhance our comparative analysis, these datasets were integrated with the 16S rRNA gene sequencing data collected from the northern NER of this study. Utilizing UPARSE 7.1, the sequences were clustered into operational taxonomic units (OTUs) based on a 97% sequence similarity threshold. For each OTU, the most representative sequence in terms of abundance was chosen, following the methodology established previously.

### 2.5 Diversity and statistical analyses

Cluster analysis was performed using a similarity matrix with Primer 6 software (PRIMER-E, Plymouth, UK), employing group-average linking methods and principal co-ordinates analysis (PCoA). Environmental parameters were screened using Variance Inflation Factor (VIF) analysis. Canonical correspondence analysis (Friedline et al., 2012) was performed using Past-3 software to assess the influence of environmental parameters on bacterial and archaeal community structure.^4^

Molecular Ecological Network Analysis Pipeline (MENAP) was used to construct ecological association networks (MENs) through Random Matrix Theory (RMT)-based methods to explore interactions between bacterial and archaeal communities (Deng et al., 2012).^5^ In this study, we use the network building process given by the NEMAP creator for analysis and select “Regress Poisson distribution only” to ensure that the type of network belongs to Poisson distribution. The parameters used to construct the network were shown in Supplementary Table 2. In this study, we choose the connectors (zi ≤ 2.5, Pi > 0.62) as the keystone in the community (Deng et al., 2012; Berry and Widder, 2014). We used the Kruskal-Wallis rank sum test, FDR multiple testing correction, and the Tukey–kramer Post-hoc test (0.95) to resolve differences between each group of species (Schloss et al., 2009).

Prediction of metabolic pathways based on 16S rRNA amplicon data was performed using FAPROTAX, which is a database that maps prokaryotic taxa to metabolic or other ecologically relevant functions based on representative literature in culture (Deng et al., 2012; Louca et al., 2016).

## 3 Results

### 3.1 Physico-chemical characteristics

In this study, 35 water samples from the Ninety-East Ridge (NER), collected at five CTD stations, were subjected to metabarcoding analysis. A depth-wise gradient of samples was established ranging from the DCM to the bathypelagic water (Figure 1). The collected 35 samples were categorized into three distinct groups based on depth and DO levels: the upper water layer (designated as NER_UP), the middle water layer (NER_MID), and the deep water layer (NER_DOWN). The physicochemical parameters for these samples are detailed in Supplementary Table 1 and Supplementary Figure 1.

### 3.2 Alpha diversity and beta diversity of microbial communities

The sequencing process yielded 1,558,711 bacterial sequences and 1,473,095 archaeal sequences from 35 individual datasets. Amplicon Sequence Variants (ASVs), determined using a 99% similarity threshold, were counted, with bacterial ASVs ranging from 16,437 to 44,104 and archaeal ASVs ranging from 17,999 to 51,229. Alpha diversity assessment across the 35 samples indicated near-complete coverage of the microbial communities, supported by rarefaction curves that approached a plateau, thereby suggesting that the sampling depth was sufficient (Supplementary Figures 2 –5). Further analysis of alpha diversity revealed that bacterial communities exhibited greater richness and diversity than archaeal communities, with NER_UP showing higher species diversity than NER_DOWN (Supplementary Figures 4, 5). In contrast, archaeal communities displayed higher richness and diversity in the NER_MID compared to both the upper and deeper layers (Supplementary Figure 5).

**Figure 2:**
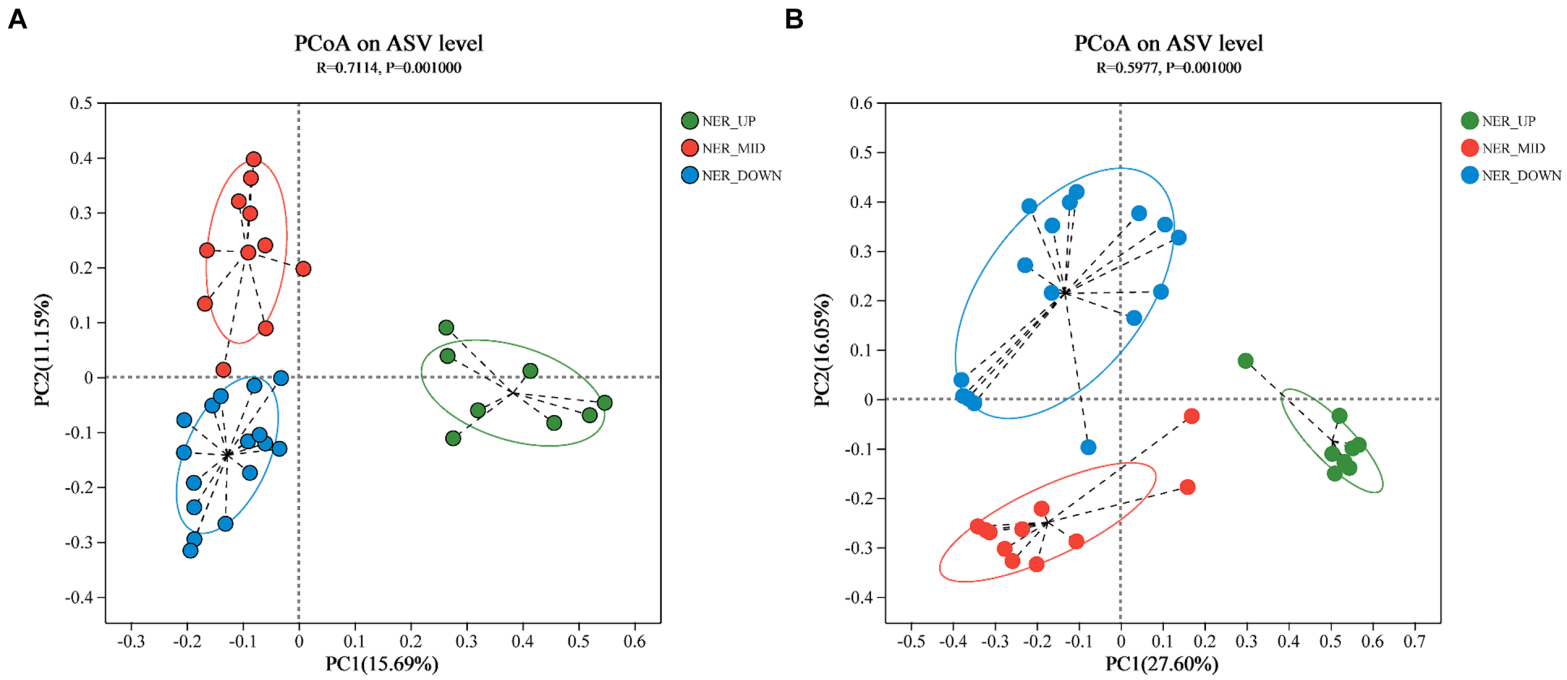
PCoA plot based on Bray-Curtis distance indicating the grouping of samples. **(A)** Bacterial communities at ASV level. **(B)** Archaeal communities at ASV level.

Principal Coordinates Analysis (PCoA) was employed to elucidate the differences among bacterial and archaeal communities at the ASV level (Navas-Molina et al., 2013). The analysis yielded R-squared (*R*2) values of 0.7114 for bacteria and 0.5977 for archaea, with corresponding *p*-values (ANOSIM) of 0.001 in both cases. These results indicated a greater degree of dissimilarity between rather than within community groups. Distinct depth layers (UP, MID, and DOWN) exhibited separate clustering for both bacterial and archaeal communities, as illustrated in Figure 2.

### 3.3 Microbial community composition in the NER water column

This study conducted an in-depth analysis of the microbial community within the NER water column, comparing their compositions across three distinct depth-related groups. The bacterial sequencing data identified 28 shared phyla, 252 genera, and 236 ASVs across the 35 water samples (Supplementary Figure 6). For archaeal communities, 16 genera and 17 ASVs were consistently present among the samples (Supplementary Figure 7).

**Figure 3:**
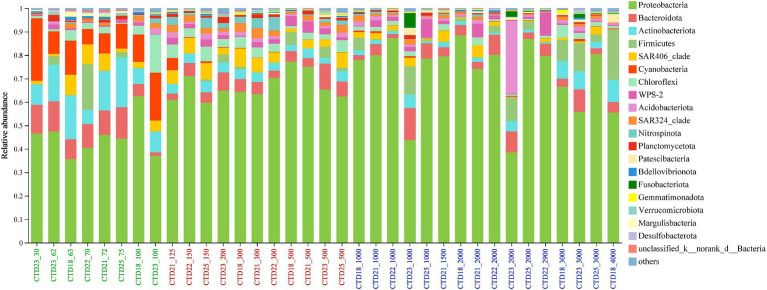
Microbial community composition at phylum level in northern NER.

The predominant bacterial phyla included *Proteobacteria*, *Bacteroidota*, *Actinobacteriota*, *Firmicutes*, SAR406 clade, *Cyanobacteria*, *Chloroflexi*, *Acidobacteriota*, and SAR324 clade. These members were dominant across nearly all samples, though their relative abundances varied (Figure 3). *Proteobacteria*, notably dominated in all samples, peaking at 88.49% in CTD18_2000m sample and reaching its lowest abundance of 35.64% in CTD18_63m. The Kruskal-Wallis H test results showed that *Proteobacteria* were significantly more abundant in the deep-water layer (NER_DOWN) than in the upper layer (NER_UP). *Bacteroidota* displayed high relative abundance across all samples, while *Actinobacteriota* and *Cyanobacteria* were more prevalent in the upper layer. The SAR406 clade was more abundant in the upper and middle layers compared to the lower layers, peaking at 8.75% in CTD18_63. Among archaea, *Crenarchaeota* (8.54–89.94%) was the most dominant phylum, followed by *Thermoplasmatota* (5.02–89.30%) and *Halobacterota* (0–12.12%) (Figure 3 and Supplementary Figure 10).

At the class level, *Alphaproteobacteria* (16.55–71.91%) and *Gammaproteobacteria* (9.32–53.73%) were the most dominant classes across NER samples, with *Alphaproteobacteria* consistently more abundant than *Gammaproteobacteria*. *Bacteroidia*’s relative abundance varied from 1.43 to 13.60% without significant variation across the groups. *Cyanobacteriia* was more dominant in the NER_UP group (6.55–26.63%) and nearly undetectable in the NER_MID and NER_DOWN groups. *Acidimicrobiia* was also more abundant in the NER_UP group, with a significant decrease in abundance below 1,000 meters. SAR324 and *Nitrospinia* were more abundant in middle water layers. The main archaeal classes, *Nitrososphaeria* and *Thermoplasmata* accounted for over 90% of the total, with *Nitrososphaeria* more prevalent in the middle and deep layers, and *Thermoplasmata* in the upper layer (Supplementary Figures 8, 10).

At the family level, *Sphingomonadaceae* (1.0–32.4%) and *Beijerinckiaceae* (0.32–42.9%) were the most dominant families in NER, followed by *Rhodobacteraceae* (1.4–35.4%), *Alcanivoracaceae* (0–40.3%), SAR406 (0–8.41%), *Flavobacteriaceae* (0.65–10.44%), and *Cyanobiaceae* (0–26.5%). *Sphingomonadaceae* exhibited higher relative abundance in the deep sea (NER_DOWN) with a broad distribution across samples. *Beijerinckiaceae* increased in abundance with depth in NER_DOWN. *Rhodobacteraceae* was more abundant in NER_MID and NER_DOWN communities. *Alcanivoracaceae* was mainly detected in CTD21 and CTD22, with the highest abundance in CTD22-150 (40.29%) and CTD22-300 (39.67%). *Flavobacteriaceae* showed higher abundance in the upper layer (NER_UP, 3.50–10.45%), but was also notable in CTD23-500 (8.01%). *Cyanobiaceae*, a bioindicator for NER_UP (6.55–26.47%), was barely detected in NER_MID and NER_DOWN. Other families like *Microtrichaceae*, SAR86 clade and OCS116 clade were mainly distributed in NER_UP, while *Alcanivoracaceae*, *Nitrospinaceae*, *Vicinamibacterales* and SAR324 were enriched in NER_MID. In NER_DOWN, *Sphingomonadaceae* and *Beijerinckiaceae* exhibited higher relative abundance. Among archaea, *Nitrosopumilaceae* (13.20–89.4%), was the most dominant family, distributed across all depths except CTD23_30. Marine Group II (3.9–73.8%), showed higher relative abundance in the upper layer (Supplementary Figures 9, 10).

**Figure 4:**
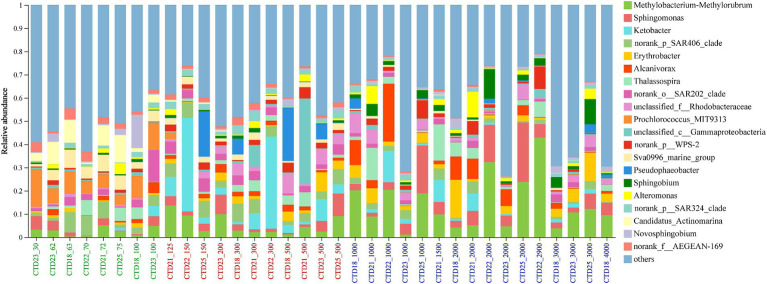
Microbial community composition at genus level in northern NER.

At the genus level, the top 10 most abundant genera included *Methylobacterium-Methylorubrum* (0.3–42.9%), *Ketobacter* (0–40.3%), *Sphingomonas* (0.3–25.6%), SAR406 clade, *Erythrobacter* (0.2–16.5%) and *Alcanivorax* (0–24.9%), *Thalassospira* (0–15.65%), SAR202 clade (0–13.8%), unclassified *Rhodobacteraceae* (0–8.5%) and *Prochlorococcus* MIT9313 (0–16.26%). *Methylobacterium-Methylorubrum* was detected in all samples, with its highest abundance in CTD22-2900 (42.91%). *Sphingomonas* peaked in CTD25-2000 (25.62%) while *Ketobacter* was primarily found in the middle layer, particularly abundant in CTD22-150 and CTD22-300 (Figure 4). Among archaeal genera, *Nitrosopumilaceae* and Marine Group II were dominant, followed by *Candidatus Nitrosopelagicus*, Marine Group III and *Nitrososphaeraceae*. *Nitrosopumilaceae* was predominantly found at depths greater than 125 m, whereas *Candidatus Nitrosopelagicus* was primarily identified in depths shallower than 150 m, which suggesting a broader ecological range for *Nitrosopumilaceae* across these samples.

### 3.4 Bioindicator taxa in the water column in NER

**Figure 5:**
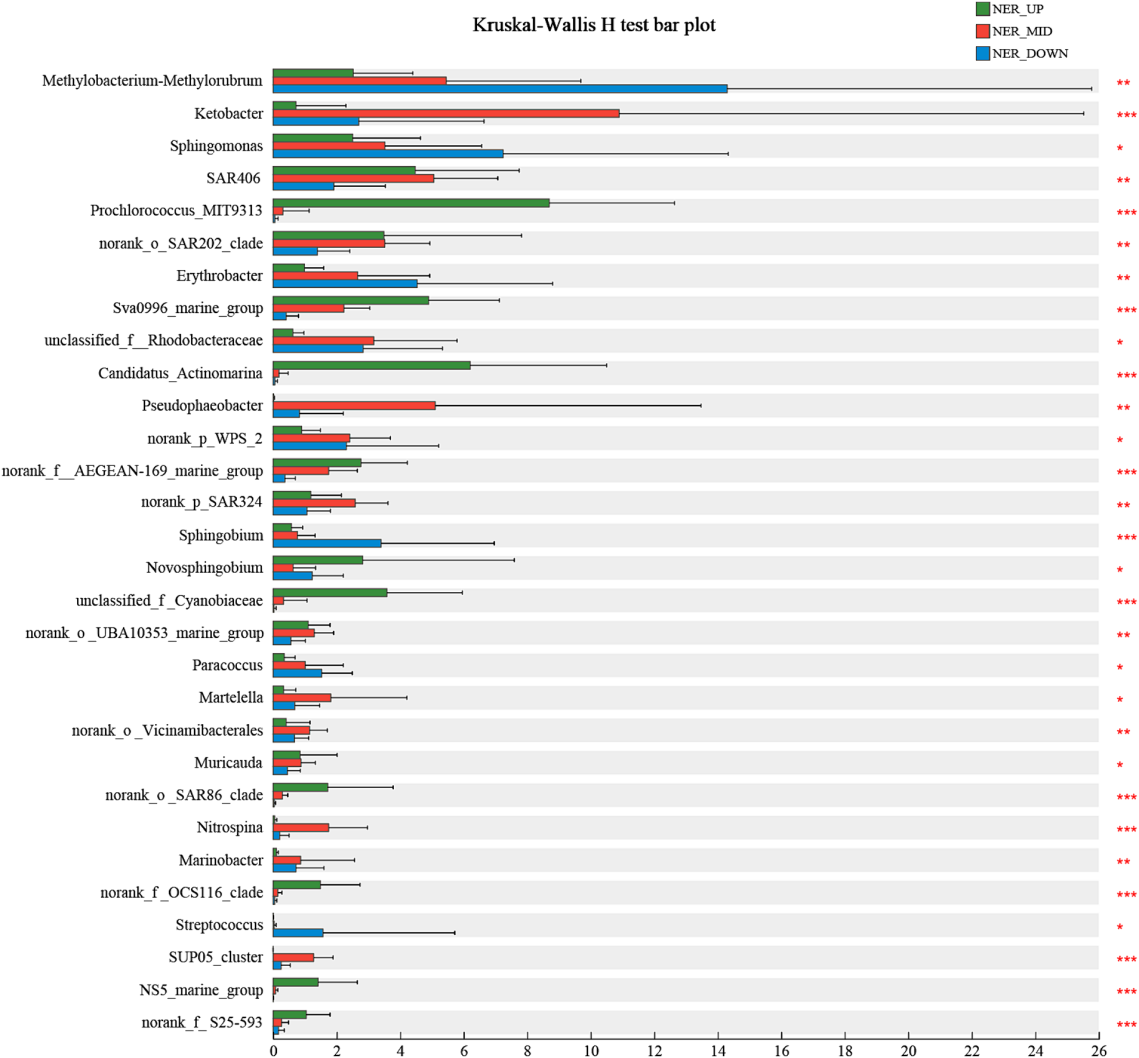
The bioindicator taxa that significantly differentiate NER_UP, NER_MID, and NER_DOWN communities in NER.

The microbial community composition was further analyzed across various water layers of the NER to identify indicator species that elucidate the relationship between microbial communities and their habitats. Using the Kruskal-Wallis H test, at least 30 bioindicator taxa at the genus level were identified, each with significant distribution patterns that distinctly differentiate the microbial communities within the upper (NER_UP), middle (NER_MID), and deep (NER_DOWN) water layers. In the NER_UP layer, bioindicator taxa included *Prochlorococcus* MIT9313, Sva0996 marine group, *Candidatus Actinomarina*, AEGEAN-169 marine group, *Novosphingobium*, *Cyanobiaceae*, *Candidatus Nitrosopelagicus*, SAR86 clade, OCS116 clade, NS5 marine group and S25-593. The NER_MID layer was characterized by bioindicators such as *Ketobacter*, *Pseudophaeobacter*, SAR324, *Martelella*, *Nitrosopumilaceae*, *Nitrospina*, and the SUP05 cluster. The deep layer (NER_DOWN) featured bioindicator taxa such as *Methylobacterium-Methylorubrum*, *Sphingomonas*, *Erythrobacter*, *Sphingobium* and *Streptococcus* (as shown in Figure 5 and Supplementary Figure 11).

### 3.5 Co-occurrence patterns of microbial community in NER

To further explore the interactions within the microbial community, a co-occurrence network analysis was conducted (Supplementary Figure 12 and Supplementary Table 2). Each node in the network represents an ASV, and the size of the node is proportional to its degree of association. Connections between nodes indicate strong and significant correlations in abundance (|r| > 0.725, *p* < 0.01). Keystone taxa, which have a significant influence on the community’s structure and function, were identified using established criteria (zi ≤ 2.5, Pi > 0.62) (Deng et al., 2012). The bacterial keystones included ASV69 (*Alcanivoracaceae*), ASV165 (*Alteromonadaceae*), ASV407 (*Flavobacteriaceae*), ASV223 (*Hyphomonadaceae*), ASV210 (*Marinobacteraceae*), ASV237 (*Pseudomonadaceae*), ASV10 (*Rhodobacteraceae*), ASV28 (SAR324 clade), ASV225 (SAR406 clade) and ASV1456 (*Sphingomonadaceae*). For the archaeal component, keystone taxa were represented by ASV168 (Marine Group II) and ASV452 (*Nitrosopumilaceae*), as detailed in Supplementary Table 2.

### 3.6 Distribution patterns of key taxa in different marine regions

**Figure 6:**
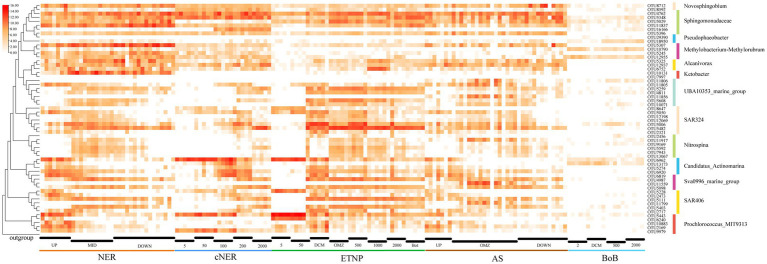
Heatmap displaying the distribution and relative abundance (log-transformed) of major OTUs for key taxa, as well as their taxonomic classification in different marine regions.

The spatial and depth-wise distribution patterns of high-abundance, bioindicator and keystone taxa were analyzed at the OTU level in the northern NER. This analysis was further extended to include comparisons with other marine regions, including the central NER (cNER), the eastern tropical North Pacific Ocean (ETNP), the Bay of Bengal (Bob), and the Arabian Sea (AS) (Figure 6).

*Methylobacterium-Methylorubrum*, a prominent bioindicator, was markedly enriched in the middle and deep water layers of the northern NER, represented by at least three dominant OTUs: OTU5307 (*Methylobacterium mesophilicum* JCM2829), OTU12955 (*Methylobacterium indicum* SE2.11) and OTU5245 (*Methylorubrum populi* BJ001). These taxa exhibited lower relative abundance in the cNER, ETNP, BoB and AS, indicating a unique distribution within the NER. Similarly, the genus *Ketobacter*, another key bioindicator, was significantly enriched and almost exclusively predominant in the northern NER’s middle and deep layers. However, it was found in low abundance or absent in the cNER, ETNP, BoB, and AS, suggesting a preference for the unique environmental conditions of the NER. *Erythrobacter* (represented by OTU4762) and *Sphingobium* were dominant in the northern NER, with *Erythrobacter* showing a wide distribution across the cNER, ETNP, and AS, especially enriched in the middle and deep water layers. *Sphingobium*, primarily found in the deep water layer of the northern NER, with OTU5348 and OTU5029 being particularly notable. They were also abundant in the deep water layer of the ETNP and AS, but exhibited lower relative abundance in the BoB, with OTU5029 almost undetected in the cNER. *Sphingomonas*, another deep-water bioindicator in the northern NER, was represented by OTU11837 (*Sphingomonas paucimobilis* NBRC 13935) and OTU5396 (*Sphingomonas suaedae* XS-10), with a distribution limited to regions other than the BoB. *Alcanivorax*, represented by at least three abundant OTUs: OTU5325 (*Alloalcanivorax dieselolei* B-5), OTU12937 (*Alloalcanivorax venustensis* ISO4) and OTU6752 (*Alcanivorax* BDAS_s NBRC102024), displayed higher relative abundance in the northern NER compared to the cNER, AS and BoB, but was also found in the ETNP. Notably, OTU5325 and OTU12937 were particularly dominant in the middle and deep layers across several regions including the northern NER, ETNP and AS. *Nitrospina*, identified as a key bioindicator in the northern NER, was predominantly distributed in NER_MID, with affiliated OTUs also enriched in the ETNP and AS, represented by OTU5592, OTU11917, OTU7943, and OTU13067.

Depth-wise distribution patterns were observed in several important taxa including SAR324, SAR406, UBA10353, Sva0996 marine group, *Candidatus Actinomarina*. The SAR324 clade displayed clear ecological niche partitioning, with OTU5482 and OTU5006 prevalent below the DCM layer across regions. OTU5050 was mainly found above the OMZ but also detected at 2000 m depth in some ETNP stations. OTU12069 was observed in the middle water layers, while OTU2321 was primarily in the DCM layer. This pattern was mirrored in the cNER, ETNP, and AS. The SAR406 clade also exhibited stratified ecological niche differentiation, with OTU5111 and OTU5403 primarily below the surface layer in the NER, while OTU5228 was advantaged in the surface layer and DCM. OTU2473 and OTU2717 were mainly in the DCM layer. Similar patterns were observed in the cNER, ETNP, and AS. UBA10353, a dominant taxon in the northern NER, exhibited ecological differentiation into epipelagic and meso-bathypelagic ecotypes, with a similar distribution in the cNER, ETNP and AS. The epipelagic ecotype, represented by OTU11805, was prevalent in the upper layers of the northern NER, while the meso-bathypelagic ecotype included OTU5259, OTU5608, OTU4811, and OTU10071. The Sva0996 marine group also displayed ecological stratification, with one ecotype dominating the DCM layers (represented by OTU6819) and another prevailing in the mesopelagic and deep water layers (represented by OTU4987 and OTU5098), a pattern consistent across the northern NER, cNER, AS and ETNP. Additionally, *Candidatus Actinomarina*, recognized as a bioindicator in the upper layers, was significantly enriched across all studied regions, with OTU6962 dominating above the DCM layer especially in the surface layer, while OTU13173 and OTU5274 prevailed in DCM layer.

### 3.7 Environmental factors influencing microbial communities structure and functional predictions for microbial communities

The physical and chemical parameters of 35 seawater samples, collected from five stations in the northern NER were detailed in Supplementary Table 1 and Supplementary Figure 1. Prior to employing canonical correspondence analysis to evaluate the impact of environmental parameters on the structure of bacterial and archaeal communities, a screening utilizing the Variance Inflation Factor (VIF)^6^ was conducted (Friedline et al., 2012). This step was essential to ensure the accuracy of the CCA analysis, which involved removing depth, phosphate and temperature due to their strong interaction (Supplementary Table 3).

**Figure 7:**
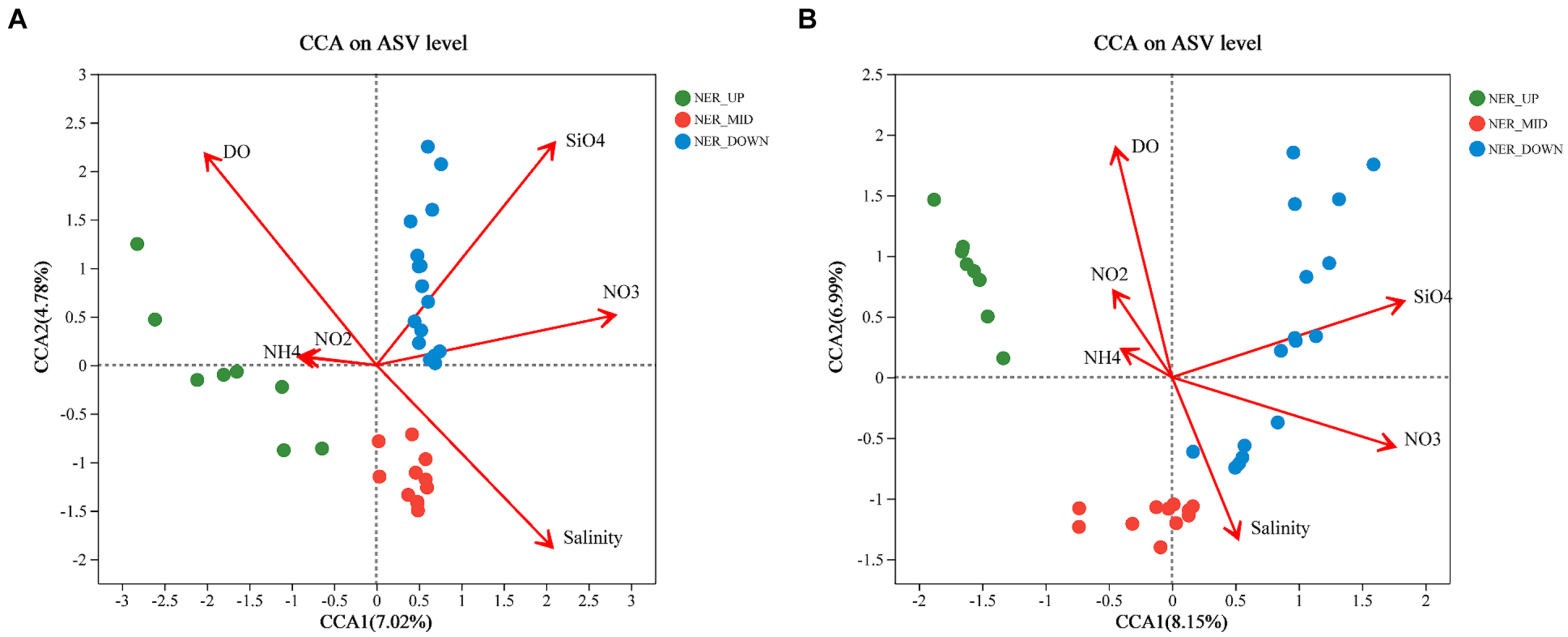
Canonical correspondence analysis (CCA) ordination diagrams of bacterial and archaeal communities: **(A)** Bacteria at the ASV leve; **(B)** Archaea at the ASV level.

The results showed that environmental factors, including DO, salinity, silicate and nitrate, were significantly correlated with bacterial and archaeal communities. For bacterial communities in the NER, the first two axes of CCA explained 11.8% of the variance at the ASV level (Figure 7). DO emerged as a key factor, distinguishing “oxygen-rich” from “oxygen-limited” groups, with all middle layer (NER_MID) samples showing a negative correlation with DO. Silicate negatively correlated with samples from the upper (NER_UP) and middle (NER_MID) layers (Figure 7). For archaeal communities in the NER, the first two CCA axes accounted for 15.14% of the total variance at the ASV level, with the NER_MID samples also showing a negative correlation with DO. The NER_UP archaeal communities were notably influenced by nitrate, while nitrite had a minor impact, indicated by its proximity to zero on the plot (Figure 7).

A spearman correlation heatmap illustrates the relationships between prokaryotes and environmental factors within the NER (Supplementary Figures 13, 14 and Supplementary Table 4). The family *Sphingomonadaceae* displayed no correlation with DO but showed significant positive correlation with silicate and negative correlation with nitrite. Genera such as *Erythrobacter*, *Alcanivorax* and *Thalassospira* exhibited significant positive correlations with nitrate (Supplementary Figure 13). Bioindicators of NER_UP, including *Prochlorococcus* MIT9313 and AEGEAN-169 marine group exhibited significant negative correlations with silicate and nitrate. Bioindicators of NER_MID, including *Ketobacter*, SAR324, and *Martelella* had significant negative correlations with DO. For NER_DOWN, bioindicators such as *Methylobacterium-Methylorubrum*, *Erythrobacter*, and *Sphingobium* demonstrated significant positive correlations with silicate and nitrate. Among archaea, *Candidatus Nitrocosmicus* showed no significant correlation with environmental factors, while *Candidatus Nitrosopelagicus* correlated positively with nitrite and negatively with silicate and nitrate (Supplementary Figure 13).

Functional predictions utilizing FAPROTAX analysis indicated that chemoheterotrophy was the most active metabolic process across the various water layers. In contrast, photoautotrophy, cyanobacteria, and photoheterotrophy processes were primarily active in the upper layer and weakened with increasing water depth. Nitrate reduction was particularly prominent in the middle layer (NER_MID) and deep layers (NER_DOWN), with a weaker presence in the deeper layers of NER (Supplementary Figure 15 and Supplementary Table 5).

## 4 Discussion

Microbial communities are pivotal in marine ecosystems, significantly influencing global biogeochemical cycles and climate processes. While existing research has concentrated on Indian Ocean OMZ regions like the Arabian Sea and the Bay of Bengal, the northern Ninety East Ridge (NER) has remained relatively unexplored. Our study fills this gap by detailing the diversity and depth-stratified distribution of prokaryotic communities within the NER’s water column. We revealed the environmental factors -particularly dissolved oxygen, salinity, silicate, and nitrate-that exert significant influence on these communities. Our comparative analysis with other regions, including the central NER, Arabian Sea, ETNP, and BOB, unveils OTU-level niche differentiation among indicator species, highlighting the biogeographical heterogeneity across marine regions. This study not only enriches our understanding of microbial diversity in the understudied NER but also provides critical insights into the complex interplay between microbial communities and their environment.

### 4.1 Physicochemical drivers of prokaryotic community distribution in NER

The distribution and structure of prokaryotic communities within the NER is shaped by a complex interplay of physicochemical factors. In this study, no significant horizontal variations in salinity and temperature were observed within the water column, corroborating previous research (Li et al., 2022). In contrast, vertical profiling revealed temperature decreased with increasing depth, while salinity was found to be lower in the upper layer (NER_UP) relative to both the mid-depth layer (NER_MID) and deep layers (NER_DOWN) (Supplementary Figure 1). The dissolved oxygen (DO) levels, consistent with previous findings in the central and southern NER, exhibited well-oxygenated conditions in the upper and deep layers (NER_UP and NER_DOWN) and lower values in the OMZs where DO levels fall below 5 mL/L (Gao et al., 2020; Li et al., 2022). The trend for DO aligns with established patterns (Millero, 2010; Al-Said et al., 2018; Bandekar et al., 2018; Li et al., 2022). Furthermore, the markedly lower nitrate concentration in the upper layer, likely due to the high primary productivity, suggests active nitrogen cycling in the surface layer (Ulloa et al., 2012). The vertical stratification of these parameters likely acts as a physical barrier to microbial dispersal, contributing to the distinct microbial communities observed at different depths.

It is well-established that the structure of microbial communities is shaped by various oceanic environmental conditions, geographical isolation, environmental gradients, and other ecological factors. Microbial communities act as key drivers, co-governed by environmental filtering and biotic interactions, in shaping the spatiotemporal distribution of marine plankton at regional and global scales (Papke et al., 2003; Boenigk et al., 2006; Moreno-Pino et al., 2016; Yan et al., 2018; Zhou et al., 2018; Wang et al., 2020). Environmental conditions and competition across temperature, oxygen gradients, and depth likely drive differences between the surface, OMZ, and deep layer bacterial communities (Zorz et al., 2019). In this study, DO, salinity, silicate, and nitrate were found to be significantly correlated with bacterial and archaeal communities, with DO being a key factor distinguishing “oxygen-rich” from “oxygen-limited” communities, particularly in the NER_MID layer (Figure 7). This pivotal role of oxygen availability underscores its critical influence on the distribution and metabolic potential of prokaryotes in the NER. Silicate, an essential nutrient for diatoms, showed a negative correlation with samples from the upper and middle layers, suggesting that the availability of this nutrient may significantly affect microbial communities composition.

The environmental factors of DO, silicate, and nitrate significantly influenced the top 10 bacterial taxa in the NER region. Similar results were found in other ocean areas regarding the prokaryotic communities in the surface, middle, and deep layers (Friedline et al., 2012; Li et al., 2018; Sebastián et al., 2021; Li et al., 2022). The observed correlations between environmental factors and specific microbial taxa, such as *Prochlorococcus* MIT9313 and the AEGEAN-169 marine group in NER_UP, and *Ketobacter*, SAR324, and *Martelella* in NER_MID, with DO and silicate, respectively, indicated that these bioindicators could reflect specific environmental conditions. These findings not only contribute to our understanding of the complex interactions governing microbial community structure in the NER but also provide a foundation for future research aimed at exploring the functional roles of these taxa in marine ecosystems and their responses to environmental changes.

### 4.2 Depth-related microbial diversity and stratification in NER

The alpha diversity indices, including the Chao1 estimator for bacterial and archaeal richness and the Shannon index for diversity, revealed a comparatively lower richness and diversity in the northern NER than in the southern NER. Despite this, the northern NER harbored a greater richness and diversity than the middle NER, a pattern potentially linked to the varying latitudes within the NER (Gao et al., 2020; Li et al., 2022). While no significant differences in bacterial richness were observed across water layers, the upper layer (NER_UP) showed higher microbial diversity than the deeper layer (NER_DOWN). In contrast, archaeal richness and diversity peaked in the middle layer (NER_MID) (Supplementary Figures 4 and 5). A total of 236 core bacterial ASVs were shared across the water column. Consistent with previous findings, the number of unique ASVs in NER_UP was significantly lower than in NER_MID and NER_DOWN (Supplementary Figure 6; Gao et al., 2020; Li et al., 2022). PCoA analysis confirmed significant separation between the three water layer groups, NER_UP, NER_MID, and NER_DOWN, underscoring depth as a pronounced differentiating factor (Figure 2). For archaea, beta diversity analysis further revealed distinct community structures across NER_UP, NER_MID, and NER_DOWN, highlighting the influence of depth on archaeal community composition. These findings indicate that depth-related environmental gradients significantly influence the stratification and structuring of microbial communities in the NER.

### 4.3 Dominant microbial taxa, their ecological differentiation and environmental adaptations

In this study, the bacterial communities across all samples were predominantly composed of *Proteobacteria*, *Bacteroidota*, *Actinobacteria*, *Firmicutes*, SAR406, *Cyanobacteria*, *Chloroflexi*, WPS-2, *Acidobacteriota*, SAR324, *Nitrospinota*, and *Planctomycetota*, which are also commonly found in diverse pelagic regions (Baldwin et al., 2005; Raes et al., 2018; Sun et al., 2021; Gu et al., 2022). The dominance of *Alphaproteobacteria* and *Gammaproteobacteria*, in particular, is well-documented across various ecosystems, such as in the Southern Ocean, South Atlantic Ocean, Large Aral Sea, Antarctic Peninsula and deep-sea hydrothermal vent deposits (Schauer et al., 2010; Milici et al., 2017; Cao et al., 2019; Shurigin et al., 2019; Zhou et al., 2022). While these dominant taxa are globally recognized, the unique environmental conditions in the northern NER, likely support a unique assemblage of microbial communities. The OTU-level niche differentiation among bioindicator species underscores the region’s potential for microbial diversity and ecological niche specialization.

*Sphingomonadaceae*, a family within the *Alphaproteobacteria*, was identified as the predominant bacterial family. Genera within this family including *Sphingomonas, Sphingobium* and *Erythrobacter* showed the highest prevalence, particularly in the deeper water layer (NER_DOWN), suggesting a possible adaptation to the NER’s bathypelagic zone conditions. In the northern NER, *Sphingomonas*, represented by OTU11837 and OTU5396, formed a dominant taxonomic group, with OTU11837 being the most abundant. The widespread distribution of this taxon in the cNER, ETNP and AS, but significantly lower relative abundance in the BoB, indicates potential environmental adaptations within the genus. Additionally, OTU16166 was predominantly found in the BOB, further highlighting the spatial heterogeneity in microbial community composition. *Sphingomonas* species are known for their metabolic versatility, a trait well-documented across various environments, including marine ecosystems, terrestrial habitats, and anthropogenic settings (Göker et al., 2017). Their ability to metabolize a wide range of naturally occurring organic compounds and to remediate a spectrum of refractory environmental contaminants is a distinctive characteristic. This metabolic adaptability is likely crucial for their ecological success, particularly in the deep ocean, where they may significantly contribute to the nutrient cycling nutrients and pollutant degradation (Balkwill et al., 2006). The genus *Sphingobium*, also a deep-water bioindicator in the northern NER, was widely distributed across water column in the cNER, ETNP and AS, with OTU5348 and OTU5029 being particularly notable. However, OTU5029 was almost undetected in the cNER. The differential distribution of *Sphingobium* taxa may reflect their distinct metabolic capabilities and ecological roles, emphasizing their adaptability and niche specialization.

The *Beijerinckiaceae* family, belonging to the ecologically diverse *Alphaproteobacteria*, encompassing a spectrum of species with varying metabolic capabilities (Tamas et al., 2013). In this study, *Methylobacterium-Methylorubrum*, represented by OTU5307, OTU12955, and OTU5245, emerged as the most abundant *Beijerinckiaceae* member, and was markedly enriched in the middle and deep water layers of the northern NER. The higher abundance of *Methylobacterium-Methylorubrum*’s at specific northern NER stations, such as CTD18, CTD22, and CTD25, suggests a preference for the environmental conditions of these depths (Supplementary Figure 9). Comparative analysis across regions revealed a widespread distribution of these taxa, but they were significantly less abundant or absent in the cNER, ETNP, Bob, and AS, indicating a distinctive distribution pattern underscores the potential for unique ecological roles and environmental adaptations within the NER’s microbial communities. This group is recognized for its ability to respire nitrate and nitrite, utilizing residual oxygen present in the upper photic zone (Tavormina et al., 2013). This metabolically plastic group, known for its facultative aerobic metabolism, utilize methanol and methylamine as carbon sources (Camargo-Neves and Araújo, 2019). Previous research in the central NER has also identified *Methyloversatilis* as one of the dominant genera, implying that methyl-containing substrates may be abundant at these sites, thereby providing a niche for the proliferation of *Methylobacterium-Methylorubrum* (Gallego et al., 2005; Park et al., 2018; Li et al., 2022). The prevalence of these taxa capable of metabolizing methylated compounds, may indicate the presence of a rich source of such substrates in the NER, potentially influencing the microbial community structure and function.

The *Alcanivoracaceae* family, stands out as another dominant group in the northern NER, with the genera *Ketobacter* and *Alcanivorax* exhibiting the highest prevalence. *Ketobacter*, notably the second most abundant genus in the NER after *Methylobacterium-Methylorubrum* and *Sphingomonas*, was significantly enriched in the northern NER’s middle and deep layers, primarily attributed to the contribution of OTU10131. This genus demonstrated a marked preference for the unique environmental conditions of the NER, as evidenced by its low abundance or absence in the cNER, ETNP, Bob, and AS. *Ketobacter* is recognized for its wide ecological distribution, thriving in both nutrient-rich photic zones and nutrient-poor deep-sea environments (Sanz-Sáez et al., 2020). Its ability to function as key alkane degrader, especially in Arctic marine settings, underscores *Ketobacter*’s crucial role in carbon cycling and ecosystem stability under challenging conditions like low temperatures and high salinity (Joye et al., 2016). The presence of *Ketobacter* in the mesopelagic and bathypelagic zones, where light is scarce and nutrients are limited, further highlights its adaptability and ecological success in the deep ocean (Rappé and Giovannoni, 2003). Its active engagement in oil-polluted environments suggests its potential in marine bioremediation, where it could significantly contribute to the mitigation of hydrocarbon contamination (Harayama et al., 2004; Yakimov et al., 2007; Kim et al., 2018; Sanz-Sáez et al., 2020).

The genus *Alcanivorax*, was also notably abundant in the northern NER, represented by at least three prevalent OTUs, OTU5325, OTU12937, and OTU6752. Specifically, OTU5325 and OTU12937 were found to dominate the middle and deep layers of the NER. Compared to other regions such as the cNER, AS and BoB, *Alcanivorax* displayed higher relative abundance in the northern NER. This taxon, a ubiquitous marine bacterium, is known for its metabolic capability to utilize oil hydrocarbons. This ability is facilitated by a diverse array of genes that enable the degradation of a wide spectrum of hydrocarbons (Schneiker et al., 2006; Kim et al., 2018). The metabolic diversity within *Alcanivoracaceae* family likely contributes to its broad substrate utilization, potentially allowing these bacteria to adapt to various environmental conditions and play a significant role in the cycling of hydrocarbons in marine ecosystems (Gallego et al., 2005; Park et al., 2018; Li et al., 2022).

*Cyanobiaceae*, recognized as a bioindicator for the upper layer (NER_UP), exhibited significantly higher relative abundance in the upper layer, ranging from 6.55 to 26.47%, and was scarcely detected in the middle (NER_MID) and deeper (NER_DOWN) layers. This stratified distribution is consistent with previous observations in the central NER (Shi and Wang, 2022). The genus *Prochlorococcus*, with OTU5443 (*Prochlorococcus* scB241 526K3) as the most abundant, was predominantly identified in the shallow water sample. Previous research confirmed that *Prochlorococcus* is primarily abundant in warm oligotrophic waters, particularly in the subtropical gyres of the Indian and western Pacific Oceans (Flombaum et al., 2013). Similar to *Cyanobiaceae*, the SAR86 clade and *Candidatus Actinomarina* were primarily distributed in NER_UP, further delineating the ecological preferences of these taxa. The SAR86 clade, one of the most abundant uncultured bacterial lineages in the surface ocean, has been found in the surface waters of the Xisha Islands, South China Sea (Dupont et al., 2012; Wang et al., 2023). Pan-genome analyses have revealed at least five distinct subgroups within the SAR86_clade, each with unique geographic distribution patterns that correlate with specific environmental parameters (Dupont et al., 2012). The presence of these bioindicators in NER_UP underscores the importance of the upper layer in shaping the microbial community structure and function within the NER ecosystem.

The SAR324_clade, recently classified as a candidate phylum, has been detected in a variety of environments, reflecting its adaptability to different marine ecosystems (Malfertheiner et al., 2022). This group was identified as a bioindicator of NER_MID, displaying ecological differentiation across water layers and marine regions. Its distribution patterns have been linked to low-oxygen conditions, as evidenced by a substantial increase in abundance in the NER_MID (Supplementary Figure 5; Aldunate et al., 2018). Comparative analysis with other regions has revealed the unique ecological niches occupied by the SAR324_clade. Specifically, OTU5482 and OTU5006 were predominantly found below the DCM layer across studied areas, while OTU5050 was situated above the OMZ, also with notable detection at the 2000 m depth in some ETNP stations. Additionally, OTU12069 was observed in the middle water layers, and OTU2321 was primarily in the DCM layer, patterns that were consistent with observations in the cNER, ETNP, and AS. The SAR324 clade’s metabolic diversity, featuring both heterotrophic and autotrophic pathways, allows it to adapt to various depths and conditions (Boeuf et al., 2021; Malfertheiner et al., 2022). It has been identified as a keystone taxon in the NER, playing a crucial role in maintaining the microbial community’s structure and function, similar to its role in other marine areas like the Andaman Sea and the eastern BoB (Guo et al., 2022).

The SAR406 clade, commonly associated with OMZs and anoxic environments (Wright et al., 2013; Landry et al., 2017; Mehrshad et al., 2017; Lim et al., 2023), was observed to have a notable distribution across the middle layer of NER (NER_MID). This clade’s niche differentiation is characterized by the predominance of OTU5111 and OTU5403 below the surface layer in the NER, with OTU5228 favoring the surface layer and the DCM. Additionally, OTU2473 and OTU2717 were predominantly found within the DCM layer, echoing similar patterns observed in the cNER, ETNP, and AS. This stratified distribution pattern indicates that DO levels may significantly influence the ecological preferences of the SAR406 clade, suggesting an adaptation to varying oxygen concentrations. Transcriptomic analyses have revealed how different subgroups within the SAR406 clade participate in the biogeochemical cycles of carbon, nitrogen, and sulfur (Bertagnolli et al., 2017), highlighting their functional diversity and ecological importance in marine environments. The varied responses to DO levels and the distinct ecological roles of the SAR406 clade subgroups highlight their adaptability and the potential for distinct ecological functions across different marine environments.

The taxon UBA10353, identified as a bioindicator in the upper (NER_UP) and middle (NER_MID) layers of NER, displayed significant ecological differentiation across various marine regions, including the cNER, ETNP, AS and BoB. Proposed to lead a mixotrophic lifestyle (Martínez-Pérez et al., 2022), UBA10353 is capable of both autotrophic carbon fixation through the Calvin-Benson-Bassham (CBB) cycle and heterotrophic metabolism, highlighting its adaptability in marine microbial communities. The epipelagic ecotype, represented by OTU11805, favored the upper layers of the northern NER but was absent in the BoB, suggesting a specialization in light-dependent processes. Conversely, the meso-bathypelagic ecotype, characterized by OTUs such as OTU5259, OTU5608, OTU4811, and OTU10071, was prevalent in the middle and deep layer across the northern NER, cNER, ETNP and AS, with the absence of OTU10071 in cNER. The detection of unique OTUs like OTU11856 solely in the AS suggests region-specific adaptations. These distribution patterns of UBA10353 ecotypes reflect the complex interplay between microbial communities and their environments.

Archaea are integral to the biogeochemical cycles of the global ocean, playing key roles in processes such as nitrification and carbon cycling (Herndl et al., 2005; Knittel et al., 2005; Leininger et al., 2006; Lipp et al., 2008). In the northern NER, *Crenarchaeota* and *Thermoplasmatota* emerged as the predominant phyla. *Crenarchaeota*, known for its potential role in nitrification, may significantly contribute to the oceanic nitrogen cycle, while *Thermoplasmatota* could be essential in the marine and terrestrial carbon cycles (Wuchter et al., 2006; Zheng et al., 2022). *Crenarchaeota*, particularly the family *Nitrosopumilaceae*, are known for their potential role in nitrification and are dominant in most samples from the NER, except for specific stations (CTD23_30, CTD23_2000, CTD22_2900, and CTD25_3000) (Supplementary Figure 10). Their preference for deep-sea environments, aligns with their observed abundance in the Gulf of Alaska, where they are prevalent in the OMZ and bathypelagic archaeal communities, suggesting an adaptation to low oxygen conditions (Abdulaziz et al., 2020). *Thermoplasmatota* (Marine Group II, MG II) was found in high abundance across the NER, as represented by ASV19. This finding is consistent with reports from the East Tropical Pacific and Atlantic Oceans, where MG II phylotypes exhibit a broad ecological tolerance and are detected throughout the water column (Pereira et al., 2019). The presence of *Methanococcales*, another archaeal order, in the NER’s deeper layers (NER_DOWN) indicates that factors beyond hypoxia, such as substrates availability or other environmental parameters, might influence its distribution (Chronopoulou et al., 2017).

### 4.4 Metabolic predictions and future research

Functional predictions based on FAPROTAX analysis indicated that chemoheterotrophy was the predominant metabolic process across various water layers of the study area. This observation aligns with the notion that a significant portion of microbial communities relies on the breakdown of organic matter for energy acquisition. In the upper layer, however, photoautotrophic processes, including those of cyanobacteria and photoheterotrophs, were more active, likely due to the greater availability of light. This pattern of metabolic stratification reflects the adaptation of microbes to the environmental gradients present in the NER. The depth-specific metabolic activities suggest that microbes in different regions, including the northern NER, cNER, ETNP, AS and BoB, may exhibit unique metabolic behaviors. While the FAPROTAX analysis offers a glimpse into these potential metabolic functions based on the 16S rRNA gene sequences, it is imperative to recognize that these are predictions and may not fully correspond to the microbes’ actual *in situ* activities. The discrepancy could be attributed to a range of environmental and physiological factors that shape microbial behavior. To bridge this gap and obtain a more precise understanding of microbial metabolic activities and their ecological roles, future studies should extend beyond 16S rRNA gene analysis to include metagenomic and metatranscriptomic approaches. By integrating these comprehensive data sets with detailed environmental parameters, we could expect to uncover a more accurate depiction of the interactions between microbial communities and their environment. Such an integrated approach will not only enhance our knowledge of ecosystem functioning in the NER but also provide valuable insights into similar marine systems.

## 5 Conclusion

This study conducted an in-depth analysis of the prokaryotic communities across the northern Ninety East Ridge (NER) in the Indian Ocean, unveiling a unique microbial ecosystems with potential contributions to marine biogeochemical cycles. Utilizing high-throughput 16S rRNA gene sequencing, we categorized and analyzed 35 water samples, revealing a distinct stratification within microbial communities and identifying key bioindicator groups that dominate across various water layers.

Our findings highlight the ubiquity and predominance of *Methylobacterium-Methylorubrum* in the NER, and the dominance of *Nitrosopumilaceae* among the archaeal communities. Additionally, *Ketobacter* shows unique enrichment in the middle and deep layers of the NER, and other groups such as UBA10353, SAR324 clade, SAR406, Sva0996 marine group, and Candidatus *Actinomarina* exhibit niche differentiation across different marine regions. The distribution patterns of these key taxa, influenced significantly by environmental factors like dissolved oxygen (DO), silicate, nitrate, and salinity, offer new insights into the complex interplay between microbial communities and their surroundings.

The innovation of this study lies in revealing the depth-stratified distribution of microbial communities in the NER, the identification of key unique taxa and ubiquitous taxa, and the correlation between microbial community structure and key environmental factors. These findings not only enhance our understanding of microbial diversity and adaptation in the northern NER but also contribute valuable new perspectives to global oceanographic research. Future studies, potentially employing metagenomic and metatranscriptomic approaches, will be crucial to uncover the full metabolic potential and ecological significance of these microbes. This will provide a more holistic understanding of their contribution to marine ecosystem and their response to environmental changes.

## Data Availability

The raw sequencing reads have been deposited in the NCBI Sequence Read Archive (SRA) database under the accession number: PRJNA993832, and the raw sequence data reported in this paper also have been deposited in the Genome Sequence Archive (Genomics, Proteomics & Bioinformatics 2021) in the National Genomics Data Center (Nucleic Acids Res 2022), China National Center for Bioinformation / Beijing Institute of Genomics, Chinese Academy of Sciences (GSA: CRA018851, CRA018853), which are publicly accessible at https://ngdc.cncb.ac.cn/gsa.

## Footnotes

^1^ www.qiagen.com/insights

^2^ www.vazyme.com

^3^ www.transgen.com/enzyme/226

^4^ https://folk.uio.no/ohammer/past/

^5^ http://ieg2.ou.edu/MENA

^6^ https://cran.r-project.org/web/packages/car/car.pdf
