# Supplementary material for: Unveiling the microbial diversity across the northern Ninety East Ridge in the Indian Ocean
Source: Front Microbiol. 2024 Sep 24;15:1436735. doi: 10.3389/fmicb.2024.1436735 (PMC11458393; doi:10.3389/fmicb.2024.1436735)

Supplementary Material

# Supplementary Figures

**FIGURE S1** The temperature, salinity and depth display map of the stations.

(A) Temperature of NER, (B) Salinity of NER, (C) DO of NER.


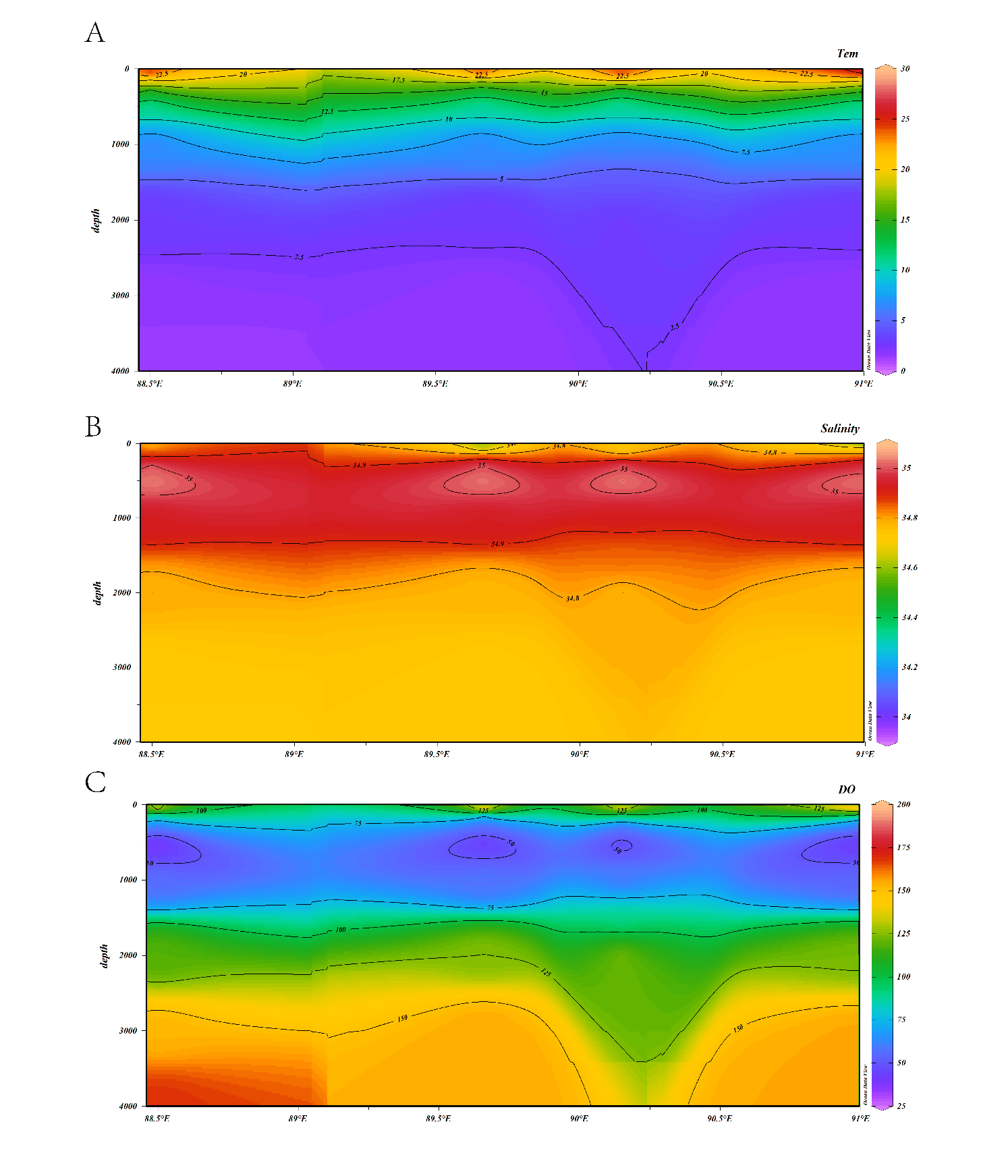


**FIGURE S2** Rarefaction curve for Bacteria.

(A) Sobs for bacteria, (B) Chao1 for bacteria, (C) Shannon for bacteria, (D) Coverage for bacteria.


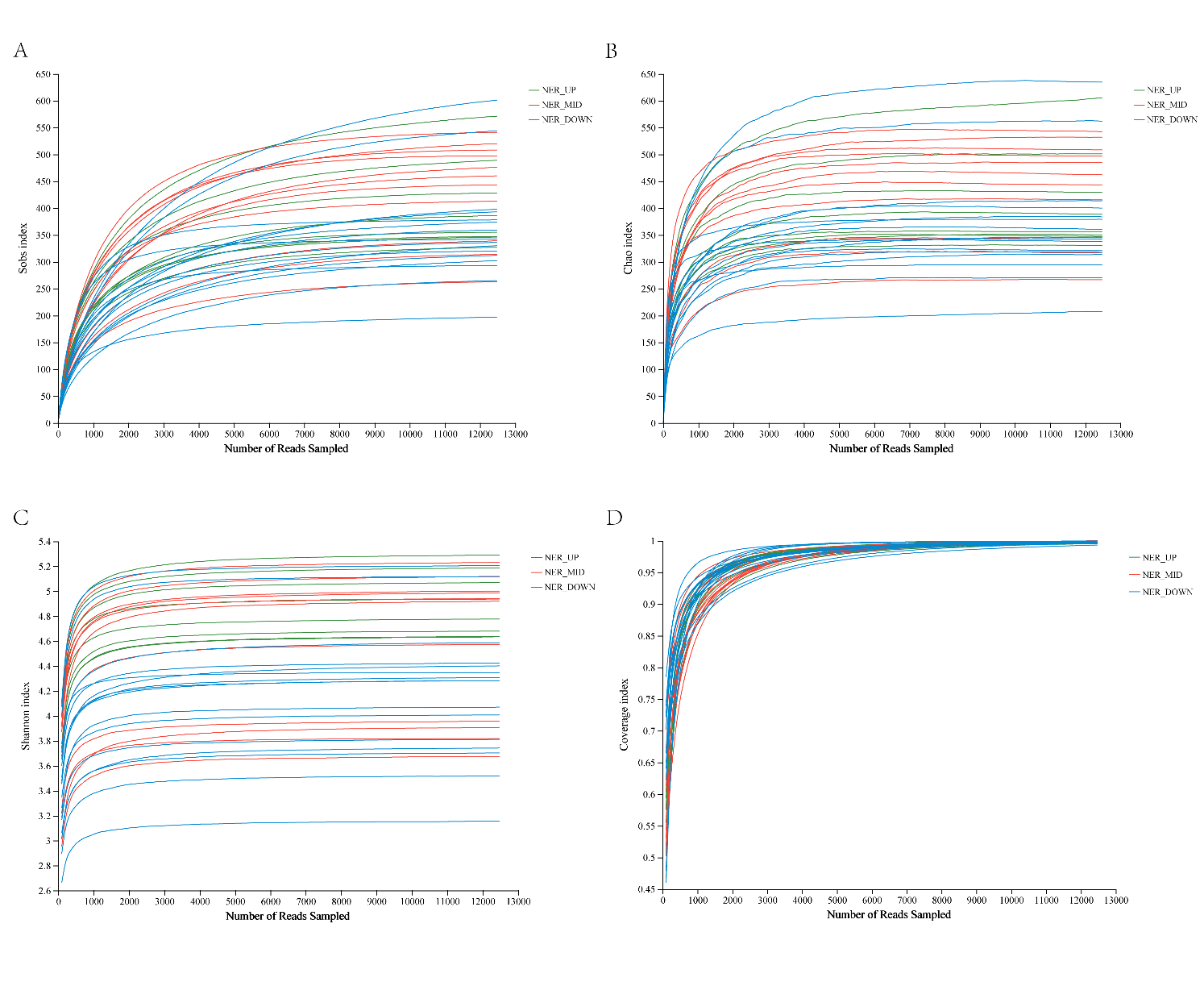


**FIGURE S3** Rarefaction curve for Archaea.

(A) Sobs for archaea, (B) Chao1 for archaea, (C) Shannon for archaea, (D) Coverage for archaea.


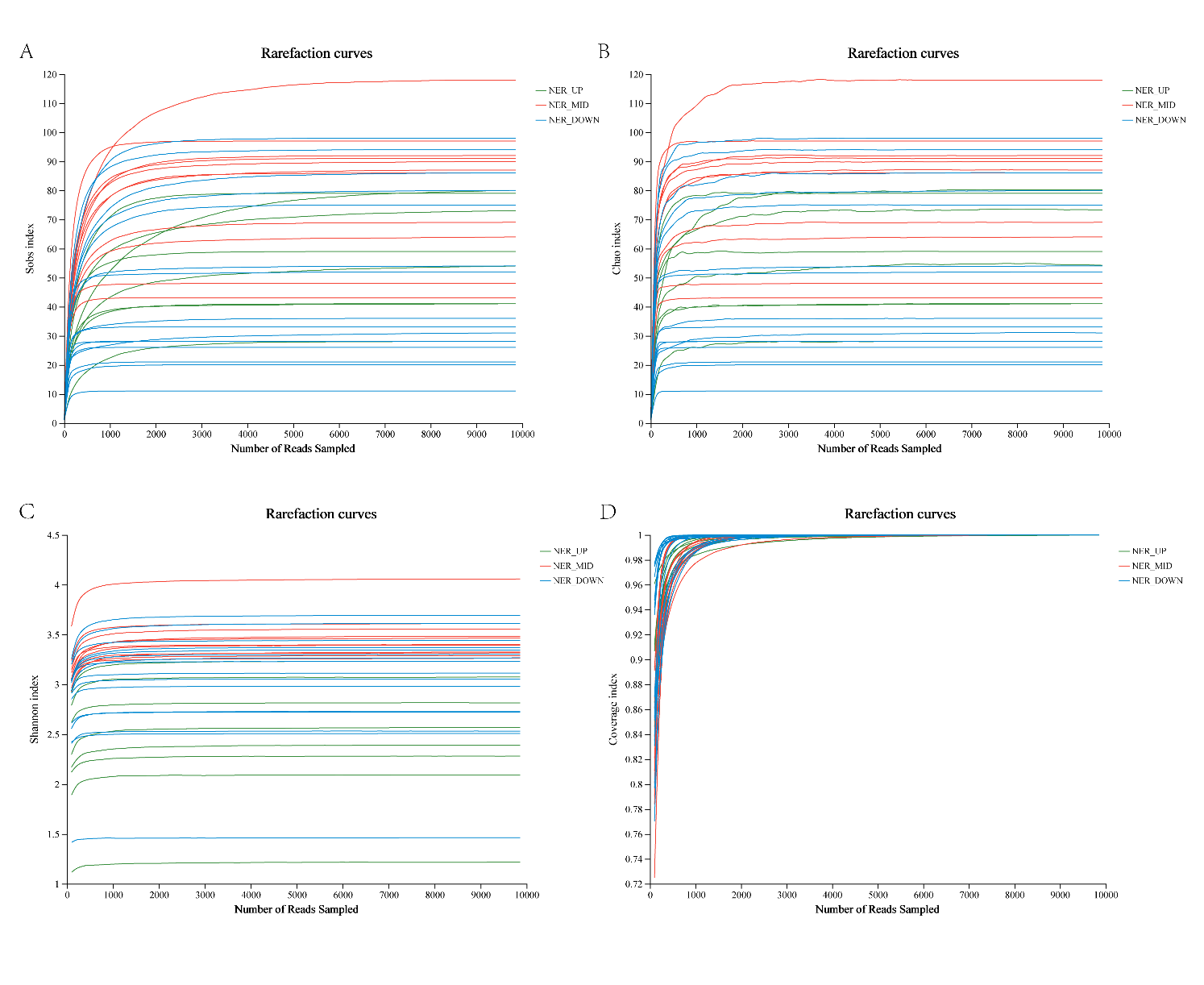


**FIGURE S4** Alpha diversity estimators for Bacteria.

(A) Sobs for bacteria, (B) Chao1 for bacteria, (C) Shannon for bacteria, (D) Coverage for bacteria.


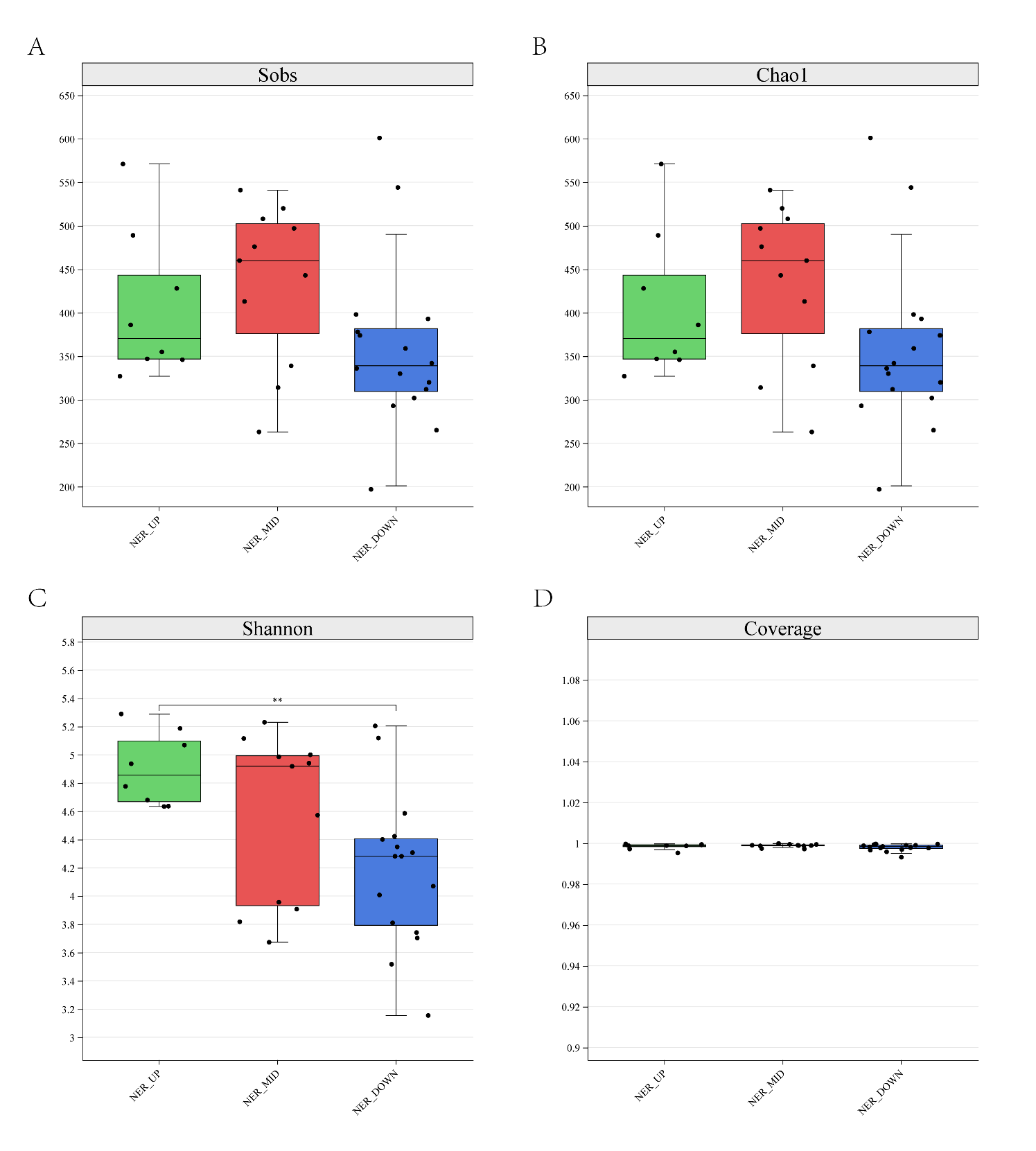


**FIGURE S5** Alpha diversity estimators for Archaea.

(A) Sobs for bacteria, (B) Chao1 for bacteria, (C) Shannon for bacteria, (D) Coverage for bacteria.


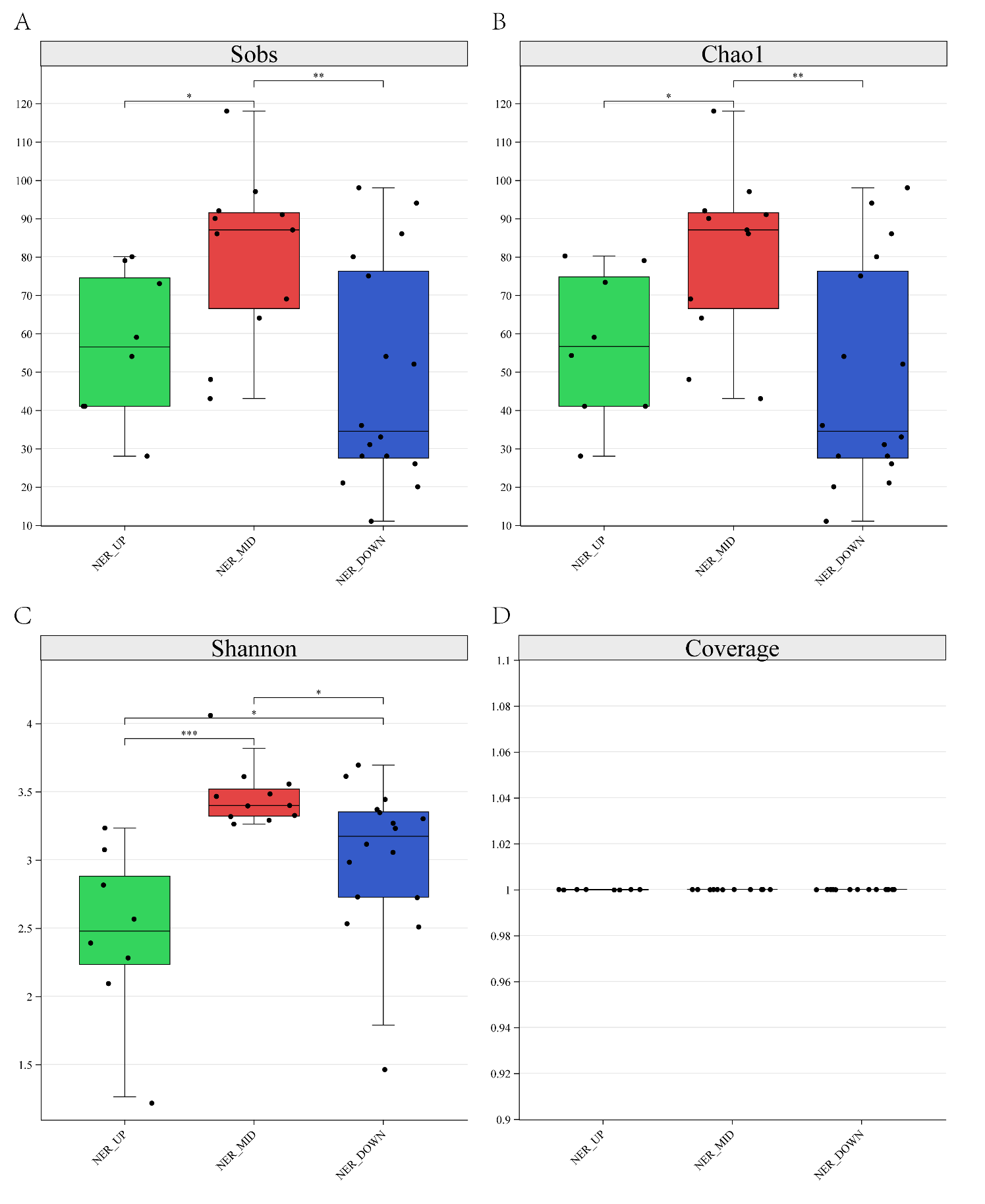


**FIGURE S6** Venn of bacterial communities.

(A) venn of bacterial at phylum level, (B) venn of bacterial at genus level, (C) venn of bacterial at ASV level.


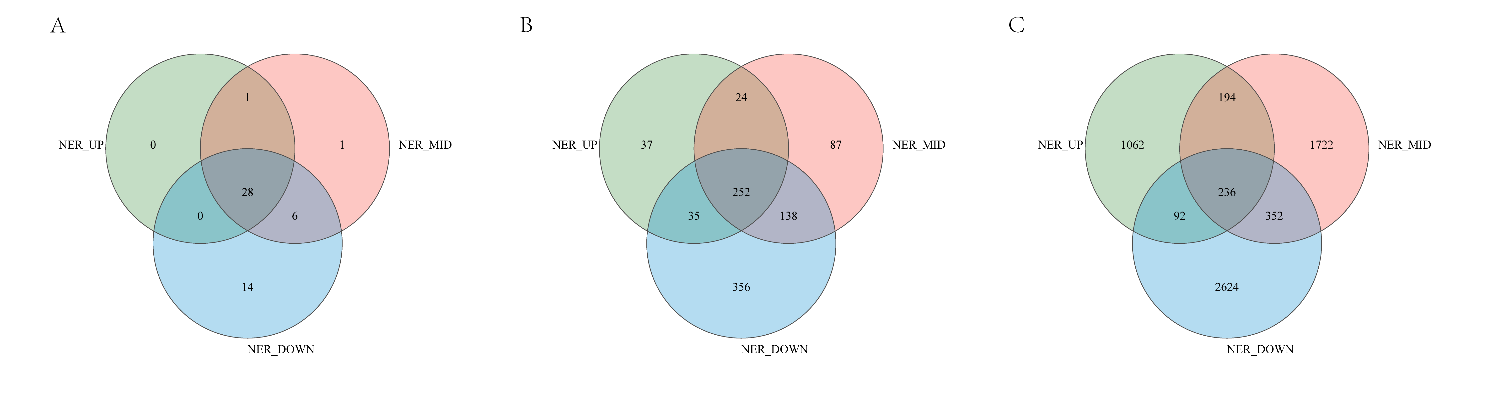


**FIGURE S7** Venn of archaeal communities.

(A) venn of archaeal at genus level, (B) venn of archaeal at ASV level.


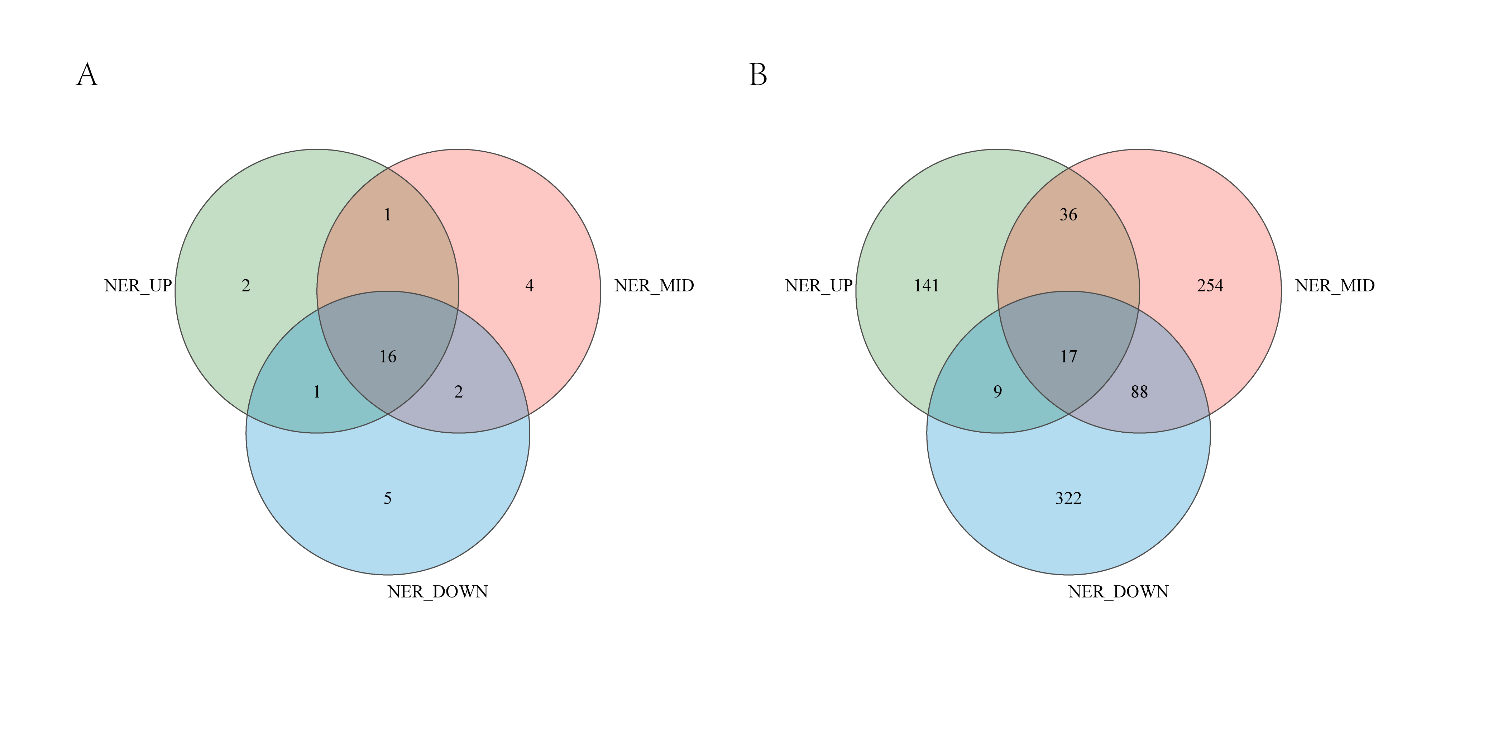


**FIGURE S8** Taxonomic distribution indicating dominant bacterial communities at class level.


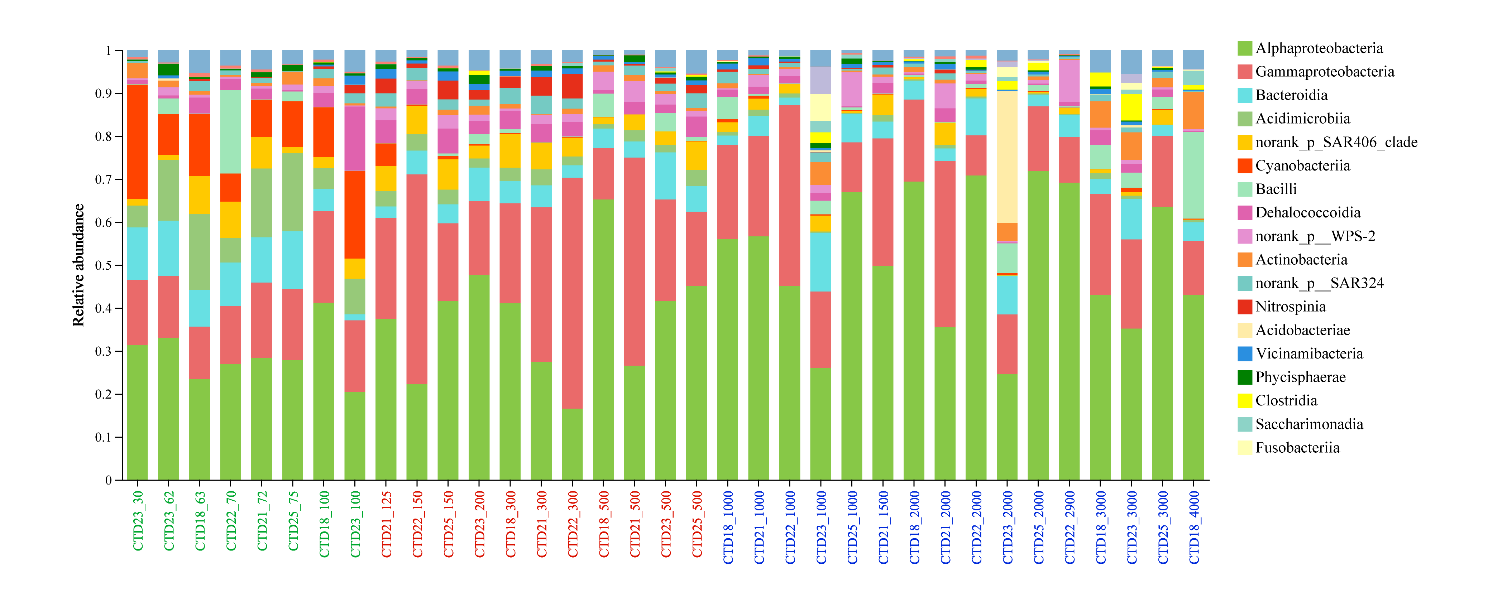


**FIGURE S9** Taxonomic distribution indicating dominant bacterial communities at family level.


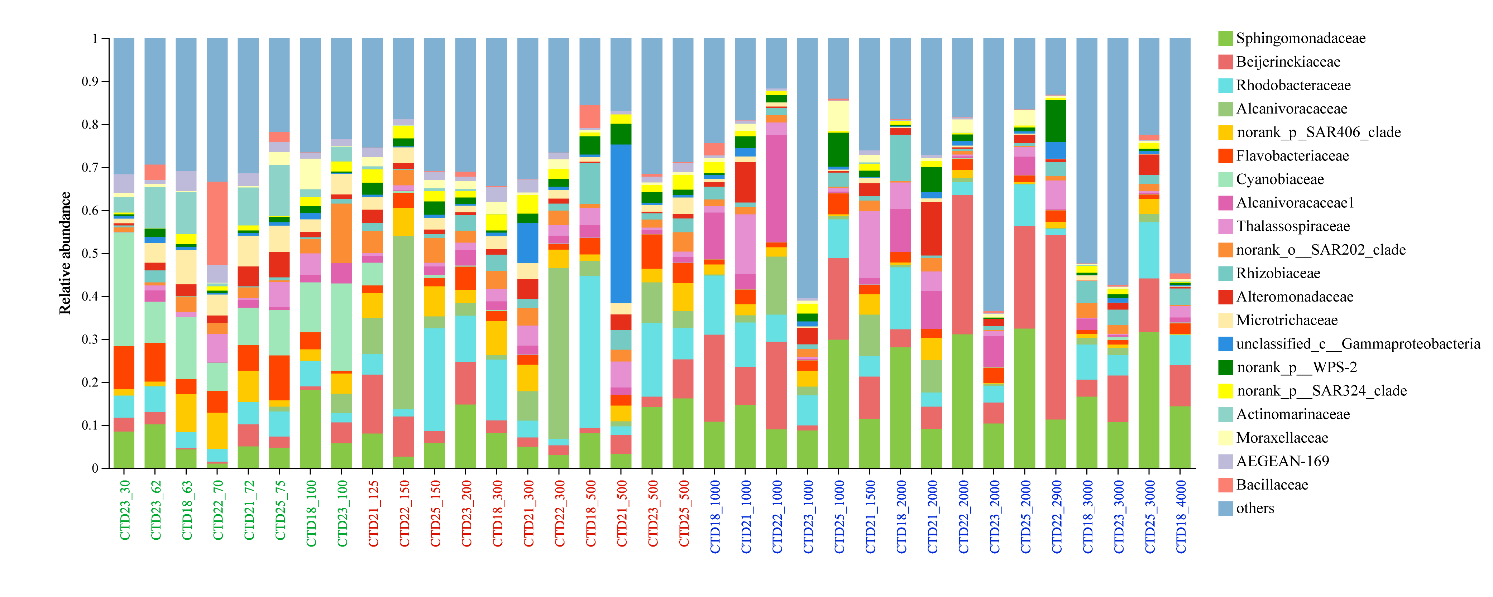


**FIGURE S10** Taxonomic distribution indicating dominant archaeal communities.

(A) archaeal at phylum level in AS, (B) archaeal at class level in NER, (C) archaeal at family level in NER, (D) archaeal at genus level in NER.


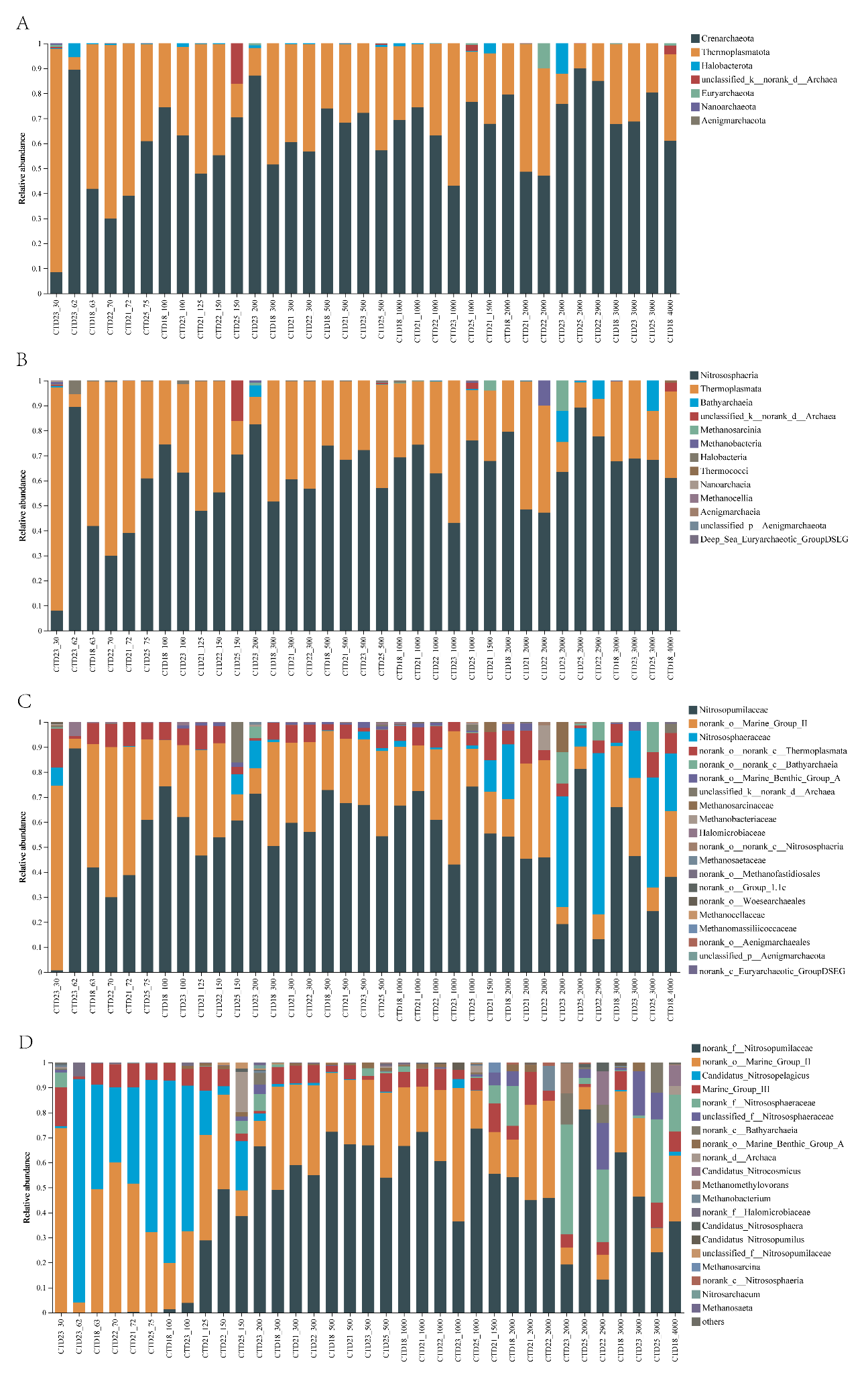


**FIGURE S11** Indicator species of archaeal.


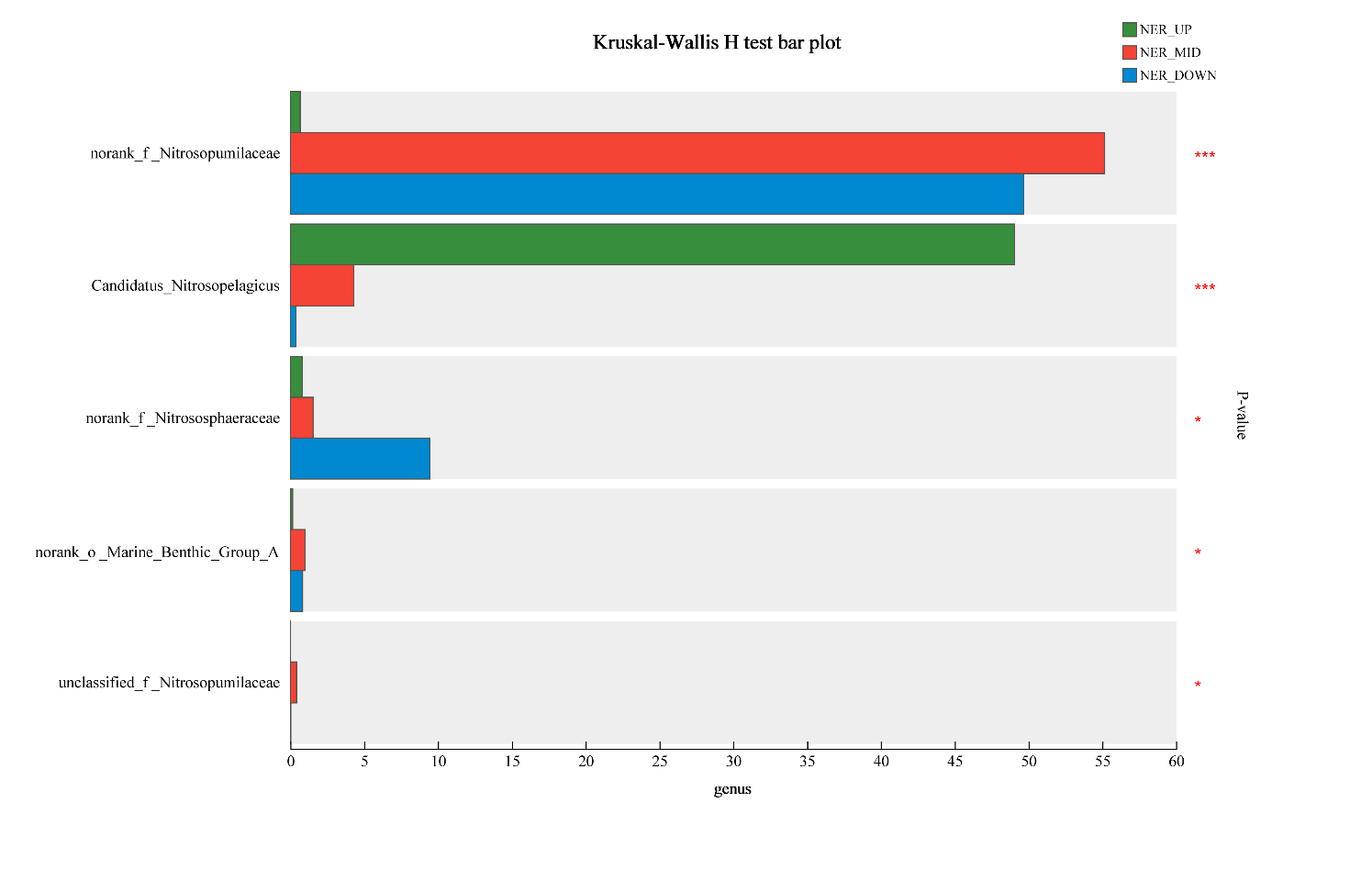


**FIGURE S12** Co-occurrence patterns of microbial populations based on Spearman’s correlation analysis.


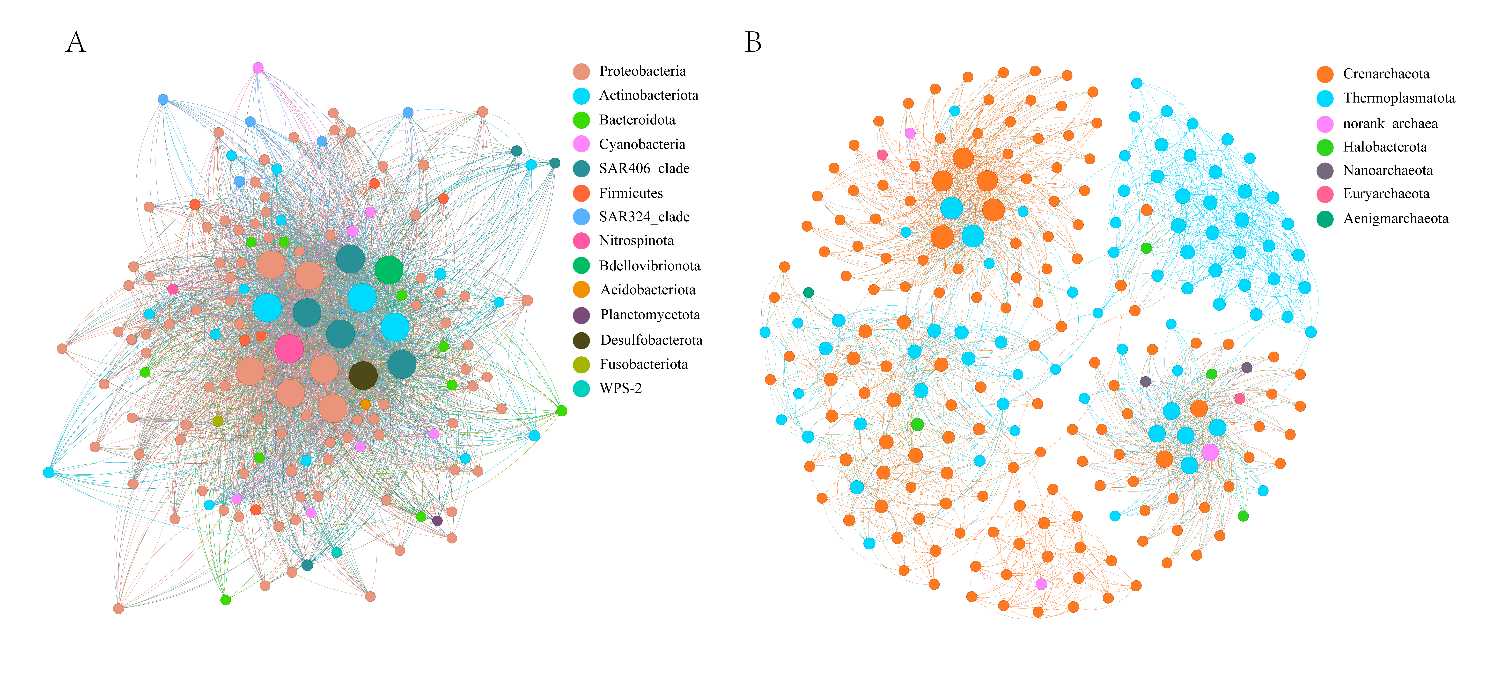


**FIGURE S13** Spearman’s correlations analysis between the environmental factors for bacterial at the genus level.


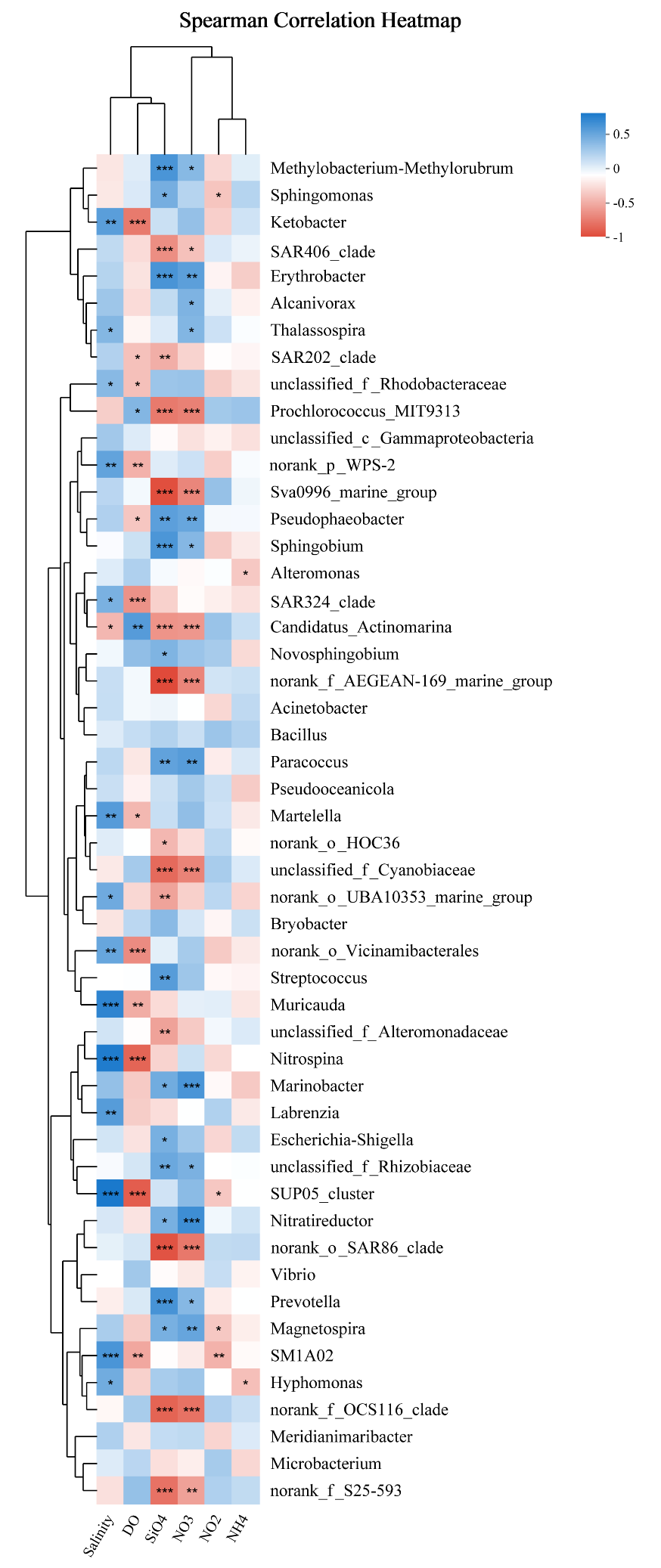


**FIGURE S14** Spearman’s correlations analysis between the environmental factors for archaeal at the genus level.


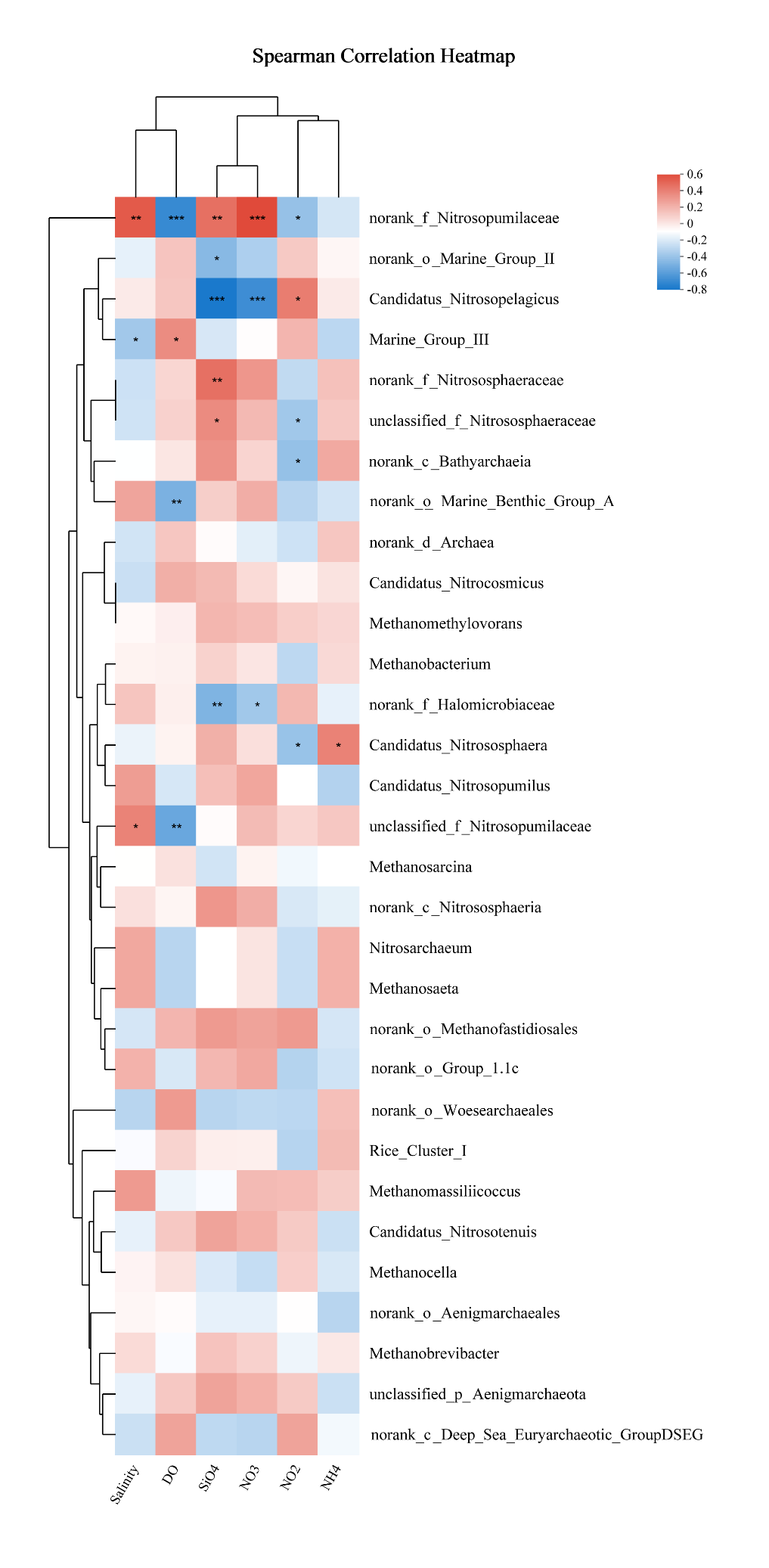


**FIGURE S15** Predictive analysis of bacterial metabolism.


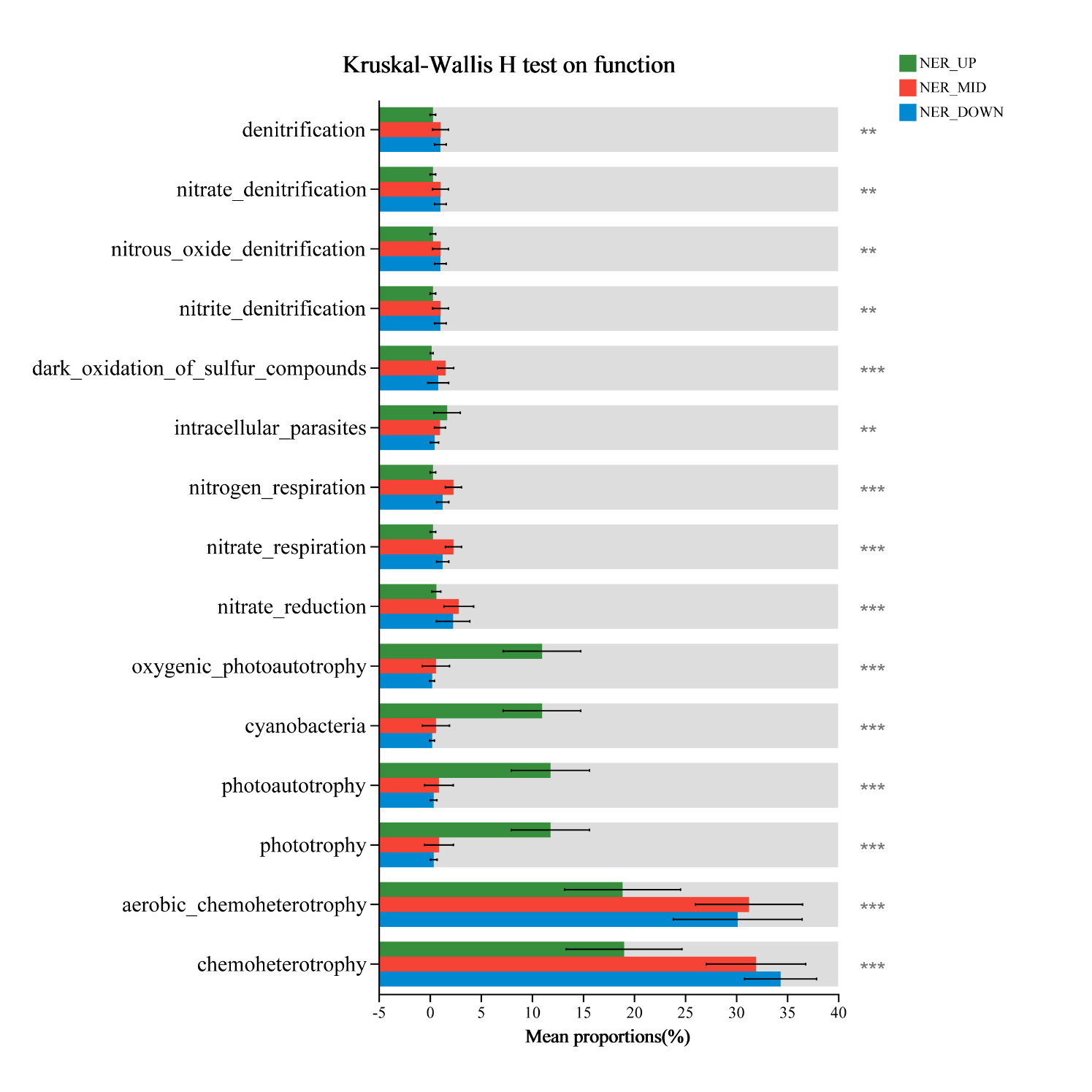

Supplement: Supplementary file 2 [file Data_Sheet_1.docx]
